# Haemoglobin values, transfusion practices, and long-term outcomes in critically ill patients with traumatic brain injury: a secondary analysis of CENTER-TBI

**DOI:** 10.1186/s13054-024-04980-6

**Published:** 2024-06-14

**Authors:** Angelo Guglielmi, Francesca Graziano, Elisa Gouvêa Bogossian, Alexis F. Turgeon, Fabio Silvio Taccone, Giuseppe Citerio, the CENTER-TBI Participants and Investigators

**Affiliations:** 1 School of Medicine and Surgery, University of Milano - Bicocca, Milan, Italy; 2 University of Pavia, PhD in Experimental Medicine, Pavia, Italy; 3 Intensive Care Department 1, Fondazione IRCCS Policlinico San Matteo, Pavia, Italy; 4 Biostatistics and Clinical Epidemiology, Fondazione IRCCS San Gerardo dei Tintori, Monza, Italy; 5 Bicocca Bioinformatics Biostatistics and Bioimaging Center B4, School of Medicine and Surgery, University of Milano-Bicocca, Milan, Italy; 6 Department of Intensive Care, Hôpital Universitaire de Bruxelles (HUB), Université Libre de Bruxelles (ULB), 1070 Brussels, Belgium; 7 CHU de Québec – Université Laval Research Center, Population Health and Optimal Health Practices Research Unit (Trauma-Emergency-Critical Care Medicine), Québec City, QC Canada; 8 Department of Anesthesiology and Critical Care Medicine, Division of Critical Care Medicine, Université Laval, Québec City, QC Canada; 9 Neurological Intensive Care Unit, Department Neuroscience, Fondazione IRCCS San Gerardo dei Tintori, Monza, Italy

**Keywords:** Haemoglobin, Anaemia, Long-term outcome, Traumatic brain injury, Blood transfusion

## Abstract

Haemoglobin (Hb) thresholds and red blood cells (RBC) transfusion strategies in traumatic brain injury (TBI) are controversial. Our objective was to assess the association of Hb values with long-term outcomes in critically ill TBI patients. We conducted a secondary analysis of CENTER-TBI, a large multicentre, prospective, observational study of European TBI patients. All patients admitted to the Intensive Care Unit (ICU) with available haemoglobin data on admission and during the first week were included. During the first seven days, daily lowest haemoglobin values were considered either a continous variable or categorised as < 7.5 g/dL, between 7.5–9.5 and > 9.5 g/dL. Anaemia was defined as haemoglobin value < 9.5 g/dL. Transfusion practices were described as “restrictive” or “liberal” based on haemoglobin values before transfusion (e.g. < 7.5 g/dL or 7.5–9.5 g/dL). Our primary outcome was the Glasgow outcome scale extended (GOSE) at six months, defined as being unfavourable when < 5. Of 1590 included, 1231 had haemoglobin values available on admission. A mean Injury Severity Score (ISS) of 33 (SD 16), isolated TBI in 502 (40.7%) and a mean Hb value at ICU admission of 12.6 (SD 2.2) g/dL was observed. 121 (9.8%) patients had Hb < 9.5 g/dL, of whom 15 (1.2%) had Hb < 7.5 g/dL. 292 (18.4%) received at least one RBC transfusion with a median haemoglobin value before transfusion of 8.4 (IQR 7.7–8.5) g/dL. Considerable heterogeneity regarding threshold transfusion was observed among centres. In the multivariable logistic regression analysis, the increase of haemoglobin value was independently associated with the decrease in the occurrence of unfavourable neurological outcomes (OR 0.78; 95% CI 0.70–0.87). Congruous results were observed in patients with the lowest haemoglobin values within the first 7 days < 7.5 g/dL (OR 2.09; 95% CI 1.15–3.81) and those between 7.5 and 9.5 g/dL (OR 1.61; 95% CI 1.07–2.42) compared to haemoglobin values > 9.5 g/dL. Results were consistent when considering mortality at 6 months as an outcome. The increase of hemoglobin value was associated with the decrease of mortality (OR 0.88; 95% CI 0.76–1.00); haemoglobin values less than 7.5 g/dL was associated with an increase of mortality (OR 3.21; 95% CI 1.59–6.49). Anaemia was independently associated with long-term unfavourable neurological outcomes and mortality in critically ill TBI patients.

Trial registration: CENTER-TBI is registered at ClinicalTrials.gov, NCT02210221, last update 2022–11–07.

**Supplementary Information:**

The online version contains supplementary material available at 10.1186/s13054-024-04980-6.

## Introduction

Traumatic brain injury (TBI) encompasses a spectrum of injuries resulting from external mechanical forces to the brain, leading to varying degrees of neurological impairment. While mild cases may present with transient symptoms, severe TBI can have profound and lasting consequences, including cognitive deficits, motor impairments, and emotional disturbances [1]. Despite advancements in emergency care and neurosurgical interventions, the long-term sequelae of TBI often pose relevant challenges for patients, caregivers, and healthcare providers [2].

Central to the management of TBI is the prevention and mitigation of secondary brain injuries, which can occur in the hours to days following the initial trauma [3]. Secondary insults, such as hypoxia, hypotension, cerebral oedema and ischemia, can exacerbate primary brain damage and significantly impact patients’ outcomes [4]. Among those, anaemia frequently coexists with TBI and has emerged as a critical determinant of patients’ outcomes [5]. The pathophysiology of anaemia in TBI is multifactorial, with contributions from acute blood loss, haemodilution, coagulopathy, and impaired erythropoiesis. Furthermore, anaemia in TBI is associated with a cascade of deleterious effects, including cerebral hypoxia, compromised tissue perfusion and increased susceptibility to secondary brain injuries [6]. Consequently, patients with TBI and concomitant anaemia often experience worse neurological outcomes, prolonged hospitalisations and increased mortality rates compared to non-anaemic TBI patients [7, 8].

The management of anaemia in TBI patients often involves administering red blood cell (RBC) transfusions to improve oxygen delivery and mitigate the adverse effects of low haemoglobin values. However, the use of RBC transfusions in this population is fraught with controversy due to concerns regarding potential complications, such as transfusion-related acute lung injury (TRALI) and increased risk of infections [9]. Moreover, emerging evidence suggests that exposure to RBC transfusion may be associated with worse outcomes in TBI patients [10]. Thus, clinicians face the challenge of balancing the potential benefits and risks of anaemia and RBC transfusions in this context, necessitating a nuanced approach tailored to individual patient factors and clinical contexts.

Several knowledge gaps and inconsistencies persist without well-conducted randomised trials in this field. Firstly, the association between anaemia and unfavourable outcomes in TBI has been frequently evaluated in single-centre studies, with significant bias on results due to local practice and the limited correction for potential confounders. Moreover, considerable heterogeneity exists in transfusion practices across different healthcare settings, with variations in haemoglobin thresholds, triggers, and strategies. Consequently, there is a pressing need for large, multi-centric studies to elucidate the potential role of anaemia as a predictive factor for unfavourable outcomes after TBI and to refine how transfusion algorithms impact these findings.

This study explored the association of Hb values with long-term outcomes in critically ill TBI patients and RBC transfusion practices.

## Methods

### Study objectives

The primary objective of this study was to evaluate whether haemoglobin values, from admission to the first week of ICU stay, were independently associated with long-term functional outcomes. Secondary objectives included the association of haemoglobin values with mortality and transfusion practices across countries.

### Study design and population

The CENTER-TBI study (NCT02210221) was a longitudinal, prospective observational study conducted across 65 centres in Europe and Israel. Detailed information regarding the study design, methodology, screening procedures, and enrolment criteria has been previously outlined [11, 12]. Within the CENTER-TBI cohort, TBI patients eligible for inclusion in this study met the following criteria: (a) aged 18 years or older; (b) admitted to the intensive care unit (ICU); (c) had at least one haemoglobin value available within 48 h from hospital admission.

The study adhered to the Strengthening the Reporting of Observational Studies in Epidemiology (STROBE) guidelines (ESM1). Research Ethics Board approval was obtained at each participating site, and all patients consented to participate in CENTER-TBI.

### Study outcomes

Our primary outcome was the Glasgow Outcome Scale Extended (GOSE), an 8-point ordinal scale measured at six months [13]. We defined an unfavourable neurological outcome as a GOSE < 5. Our secondary outcomes were mortality and the proportion of transfusions across centres.

### Data collection

Data collection and management procedures for the CENTER-TBI study have been previously outlined [11, 12]. The CENTER-TBI core database v3.0 was accessed and retrieved via the Opal data warehouse [14]. Collected data encompassed various patient parameters, including demographic characteristics, pre-existing comorbidities, mechanism of TBI and admission assessments such as neuroradiological findings [15], neurological status (e.g., Glasgow Coma Scale—GCS, pupillary reactivity), and presence of extracranial injuries (quantified by the total Injury Severity Score—ISS, with major extracranial injury defined by an Abbreviated Injury Scale—AIS score ≥ 3) [16]. Additionally, info such as neurosurgical interventions and intracranial pressure (ICP) monitoring, ICP management therapies (e.g. fluids balance) [17], and the necessity for extracranial and intracranial surgeries were collected. We didn’t have information regarding the age of RBC used for transfusion.

### Haemoglobin values

For all analyses, we refer to haemoglobin values as the daily lowest measurement of haemoglobin at ICU admission (day 1) and within the first seven days from ICU admission. Haemoglobin values were considered either a continuous variable or categorised as less than 7.5 g/dL, between 7.5 and 9.5 g/dL and above 9.5 g/dL. Anaemia was defined as a haemoglobin value less than 9.5 g/dL. Delta haemoglobin values were the difference between day 1 and day 7. According to previous literature [18–20], we defined transfusion practices as "restrictive" or “liberal” based on haemoglobin values before transfusion (e.g. < 7.5 g/dL or 7.5–9.5 g/dL).

### Statistical analysis

Descriptive analyses were conducted considering patients with haemoglobin values available at ICU admission (day 1). Transfusion practices and outcome analysis were conducted considering the overall population (patients with at least one haemoglobin value available within 48 h from hospital admission).

Continuous variables were summarised using medians and quartiles, and categorical variables using frequencies and percentages. As appropriate, comparisons between groups were performed using the Kruskal-Wallis test, t-test, Wilcoxon rank-sum test, chi-squared test, or the Fisher exact test. To evaluate the centres' and countries' variabilities, a linear mixed model was used using delta Hb, defined as the difference between the Hb values at day 7 and day 1 as an outcome and adjusted for case-mix (demographics and clinical variables, e.g. age, ISS, GCS motor score and baseline pupils’ abnormality) and centers/countries as a random effect. The variability was shown as a caterpillar plot. ANOVA test was used to compare models with and without the random effect to test if variability is significantly different. Centers or countries with less than 10 enrolled patients were allocated to the “Other” group.

Regarding the classification of “liberal” or “restrictive” among countries in transfusion, both continuous Hb values before transfusion and the proportion of the two policy types were calculated. Only centers with at least 10 or more RBC transfusions were deemed suitable for transfusion-related analyses.

The long-term outcome analysis was estimated using a logistic regression model analysis on unfavourable outcomes at 6 months (GOSE < 5) and mortality at 6 months, in which variables with a significant difference (*p* < 0.05) in the univariate analysis and clinical relevance were included (age, ISS, presence of prehospital hypotension or hypoxia, presence of any extracranial injury, GCS motor score, haemoglobin on admission, need of transfusion in the first week and weekly fluid balance). All the outcome analyses were performed considering the overall population (patients with at least one haemoglobin value available within 48 h from hospital admission). Haemoglobin, as the daily lowest haemoglobin value measured within the first seven days from ICU admission, was considered in the model as a continuous value categorised as < 7.5 g/dL, 7.5–9.5 g/dL and > 9.5 g/dL. A sensitivity analysis including only isolated TBI was also performed. The results were reported as odds ratio (OR) and corresponding 95% confidence interval (CI). A type I error rate of 0.05 was employed. All analyses were conducted using R software (version 4.0.3). 

## Results

### Study population

Among the 2006 ICU adult patients enrolled in CENTER-TBI, 1590 met eligibility criteria and were included in our study; 1231 had haemoglobin values available at ICU admission (day 1) (Supplemental Fig. S1).

The mean age of this subpopulation was 49 (standard deviation (SD) 19) years, with 918 out of 1231 (76.0%) being male. The overall mean total ISS on admission was 33 (SD 16), with 502 out of 1231 (40.7%) presenting with isolated TBI. Bilateral unreactive pupils were reported in 143 (11.0%) patients, while 170 (14.0%) experienced hypoxia, and 174 (14.2%) patients were hypotensive during transportation and/or upon arrival at the emergency department (Table 1).

**Table 1:** Characteristics of ICU patients with haemoglobin values available at ICU admission (day 1)

	Hb available on day 1 of ICU admission				*p*
	Overall (N = 1231)	< 7.5 g/dL (N = 15)	7.5–9.5 g/dL (N = 106)	> 9.5 g/dL (N = 1110)	
Haemoglobin at baseline, mean (SD)	12.6 (2.2)	6.42 (0.1)	8.7 (0.5)	13.0 (1.7)	< 0.001
Sex, female, n (%)	313 (24.0)	6 (40.0)	44 (41.5)	246 (22.2)	< 0.001
Age, mean (SD)	49 (19.)	43 (19)	55 (19)	48 (19)	0.002
Total ISS, mean (SD)	33 (16)	41 (13)	42 (18)	32 (15)	< 0.001
AIS ≥ 3					
Face, n (%)	274 (21.0)	7 (46.7)	21 (19.8)	234 (21.1)	0.051
Thorax/chest, n (%)	464 (35.6)	6 (40.0)	54 (50.9)	367 (33.1)	0.001
Abdomen/pelvis, n (%)	252 (19.3)	5 (33.3)	43 (40.6)	179 (16.1)	< 0.001
Extremities, n (%)	240 (18.4)	8 (53.3)	35 (33.3)	179 (16.1)	< 0.001
External, n (%)	44 (3.4)	0 (0.0)	5 (4.7)	33 (3.0)	0.480
Spine, n (%)	244 (18.7)	5 (33.3)	30 (28.3)	188 (16.9)	0.005
Any major extracranial injury (AIS ≥ 3), n (%)	729 (59.2)	12 (80.0)	79 (74.5)	590 (53.2)	< 0.001
Extra cranial surgery, n (%)	411 (31.7)	7 (46.7)	55 (52.4)	319 (28.9)	< 0.001
Cranial surgery, n (%)	509 (39.3)	6 (40.0)	54 (51.4)	413 (37.4)	0.019
Pupils' abnormalities, n (%)					
Both reactive	1014 (81.8)	8 (53.3)	75 (73.5)	883 (83.8)	< 0.001
One unreactive	82 (6.6)	0 (0.0)	9 (8.8)	62 (5.9)	
Both unreactive	143 (11.5)	7 (46.7)	18 (17.6)	109 (10.3)	
Glasgow coma scale, median (IQR)	9 (4–14)	6. (3–14)	6 (3–13)	10 (4–14)	0.002
GCS motor score, n (%)					
1	375 (29.4)	6 (40.0)	42 (40.8)	299 (27.5)	0.193
2	49 (3.8)	0 (0.0)	6 (5.8)	39 (3.6)	
3	57 (4.5)	1 (6.7)	5 (4.9)	48 (4.4)	
4	93 (7.3)	1 (6.7)	7 (6.8)	82 (7.5)	
5	237 (18.6)	3 (20.0)	17 (16.5)	202 (18.6)	
6	465 (36.4)	4 (26.7)	26 (25.2)	417 (38.4)	
Prehospital or ED Hypoxia, n (%)	170 (14.0)	3 (21.4)	22 (21.8)	135 (13.1)	0.039
Prehospital or ED Hypotension, n (%)	174 (14.2)	7 (46.7)	28 (28.0)	128 (12.2)	< 0.001
ICP monitoring, n (%)	551 (44.9)	7 (46.7)	60 (56.6)	484 (43.7)	0.039
Cause of injury, n (%)					< 0.001
Incidental fall	470 (39.7)	3 (20.0)	30 (29.1)	437 (41.0)	
Other	100 (8.4)	0 (0.0)	7 (6.8)	93 (8.7)	
Road traffic accident	526 (44.4)	9 (60.0)	55 (53.4)	462 (43.3)	
Suicide attempt	30 (2.5)	2 (13.3)	8 (7.8)	20 (1.9)	
Violence/assault	59 (5.0)	1 (6.7)	3 (2.9)	55 (5.2)	
Marshall CT score					
1	117 (10.7)	0 (0.0)	7 (8.0)	110 (11.1)	0.042
2	532 (48.9)	4 (36.4)	37 (42.5)	491 (49.5)	
3	83 (7.6)	2 (18.2)	8 (9.2)	73 (7.4)	
4	16 (1.5)	0 (0.0)	2 (2.3)	14 (1.4)	
5	1 (0.1)	0 (0.0)	1 (1.1)	0 (0.0)	
6	340 (31.2)	5 (45.5)	32 (36.8)	303 (30.6)	

Baseline characteristics of enrolled patients with Hb value available at day 1. Patients are categorized into three groups according to haemoglobin values at baseline: < 7.5 g/dL, 7.5–9.5 g/dL, and > 9.5 g/dL. An Abbreviated Injury Scale ≥ 3 defines major extracranial injury. Any major extracranial injury AIS ≥ 3 defines all the patients with at least one major extracranial injury in any AIS anatomical region

GCS: Glasgow coma scale; GOSE: Glasgow outcome scale extended; ISS: injury severity score; AIS: abbreviated injury scale; ED: emergency department; ICP: intracranial pressure; CT: computed tomography; SD: standard deviation. Any major extracranial injury AIS ≥ 3 defines all the patients with at least one major extracranial injury in any AIS anatomical region

### Haemoglobin values at ICU admission (day 1) and over the week

**Fig. 1:**
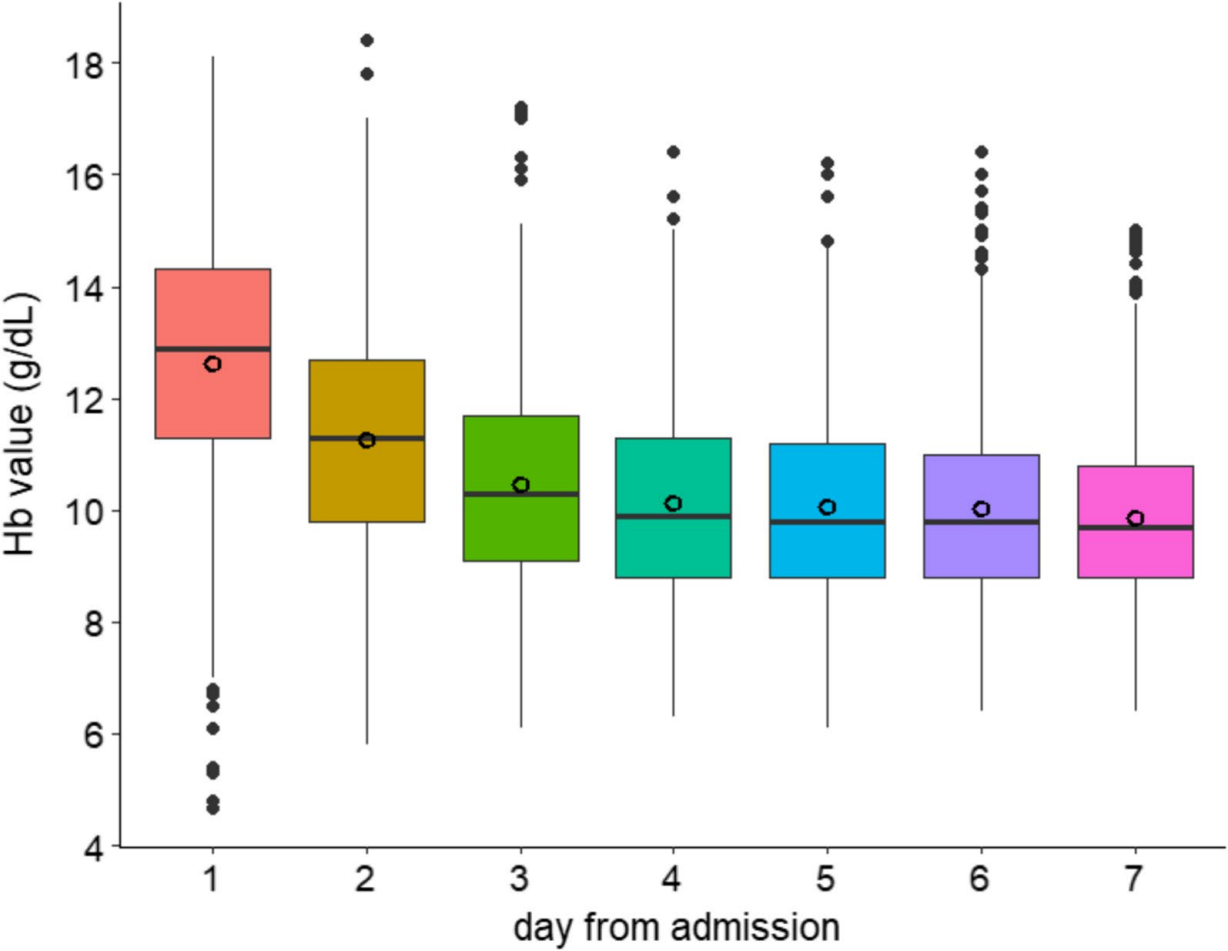
Haemoglobin values during the first week of ICU admission. Distribution of haemoglobin values during the first week of Intensive care unit (ICU) admission. The mean haemoglobin values (empty circles) gradually decreased during the first week from 12.62 (SD 2.2) to 9.8 (SD 1.5) g/dL at day 7

The mean haemoglobin value on ICU admission on day 1 was 12.6 (SD 2.2) g/dL. A total of 121 (9.8%) patients had Hb < 9.5 g/dL on admission, of whom 15 (1.2%) < 7.5 g/dL. Anaemic patients on admission presented with higher ISS scores, a higher prevalence of pupil abnormalities, more frequent episodes of prehospital hypotension and hypoxia, and a higher incidence of extracranial injury, as shown in Table 1. The mean haemoglobin values gradually decreased during the first week to 9.8 (SD 1.5) g/dL at day 7 (Fig. 1). During the first 7 days in the ICU, 762 (47.9%) patients had Hb < 9.5 g/dL and 138 (8.7%) < 7.5 g/dL (Supplemental Table 1). The proportion and the dynamics of the three categorised groups are shown in Supplemental Table 1 and Supplemental Fig. 2. The proportion of anaemic patients increased over time, while the non-anaemic patients group showed decreased Hb values at the end of the week. The proportion of the group with Hb < 7.5 g/dL remained constant over time. As shown in Supplemental Table 1, overall, patients had a positive fluid balance over the week; the highest values were found in patients with Hb < 7.5 g/dL or between 7.5 and 9.5 g/dL.

### Haemoglobin and centers/countries variability

Baseline mean values of haemoglobin amongst countries (Supplemental Table 2) ranged from 12.07 (SD 2.26) to 14.64 (SD 0.11), and amongst centres ranged from 11.00 (SD 2.30) to 14.64 (SD 0.11). Delta haemoglobin values between day 1 and day 7 across study centers and participating countries are reported in Supplemental Figs. S3A and S3B. A median negative reduction of 2.7 g/dl was observed amongst centers and countries. Following adjustment for age, ISS, GCS motor score and baseline pupil abnormality, no significant variability of countries (*p* = 0.09) or centers (*p* = 0.24) with changes in haemoglobin values over time was observed.

### Transfusion practices

292 (18.4%) of 1590 patients received at least one RBC transfusion in the first week, with 165 out of 292 patients (56.5%) receiving two or more transfusions, resulting in 562 transfusion events (Table 2, Supplemental Fig. S4). The characteristics of transfused patients can be found in Table 2. Transfused patients presented a lower GCS motor score on admission, more frequent unreactive pupils and higher ISS than others. They also experienced more frequent prehospital hypoxia, prehospital hypotension and extracranial injury. The median haemoglobin value before transfusion was 8.4 (IQR 7.7–8.5) g/dL (Table 2). There was considerable heterogeneity among countries regarding the threshold used for transfusion, as illustrated in the Supplemental Fig. S4. The Supplemental Fig. S5 depicts transfusion practices per country according to a liberal or restrictive strategy, with a prevalence of a liberal one.

**Table 2:** Characteristics of overall population ICU patients with at least one haemoglobin value available within 48 h from admission

	Hb available within 48 h from ICU admission			*p*
	Overall (N = 1590)	No RBC transfusion (N = 1298)	RBC transfusion (N = 292)	
First Hb value, mean (SD)	9.9 (1.6)	10.1 (1.6)	9.3 (1.1)	< 0.001
Mean Hb during the week, mean (SD)	10.0 (2.1)	10.4 (2.0)	8.3 (1.1)	< 0.001
Subgroup				
Weekly Hb < 7.5 g/dL	138 (8.7)	82 (6.3)	56 (19.2)	
Weekly Hb between 7.5–9-5 g/dL	624 (39.2)	415 (32.0)	209 (71.6)	
Weekly Hb > 9.5 g/dL	828 (52.1)	801 (61.7)	27 (9.2)	
Hb before transfusion, median (IQR)	–	–	8.4 (7.7–8.5)	
More than one transfusion, n (%)	–	–	165 (56.5)	
Sex, female, n (%)	392 (24.7)	309 (23.8)	83 (28.4)	0.114
Age, mean (SD)	49.0 (19.0)	49.0 (18.9)	48.7 (19.7)	0.816
Total ISS, mean (SD)	33(15.7)	31 (15)	42 (16)	< 0.001
AIS ≥ 3				
Face, n (%)	357 (22.5)	278 (21.4)	79 (27.1)	0.045
Thorax/chest, n (%)	566 (35.6)	400 (30.8)	166 (56.8)	< 0.001
Abdomen/pelvis, n (%)	292 (18.4)	182 (14.0)	110 (37.7)	< 0.001
Extremities, n (%)	293 (18.4)	195 (15.0)	98 (33.6)	< 0.001
External, n (%)	53 (3.3)	36 (2.8)	17 (5.8)	0.015
Spine, n (%)	295 (18.6)	211 (16.3)	84 (28.8)	< 0.001
Any major extracranial injury (AIS ≥ 3), n (%)	885 (55.7)	659 (50.8)	226 (77.4)	< 0.001
Extra cranial surgery, n (%)	479 (30.3)	322 (25.0)	157 (54.0)	< 0.001
Cranial surgery, n (%)	632 (40.1)	468 (36.4)	164 (56.4)	< 0.001
Pupils' abnormalities, n (%)				
Both reactive	1231 (82.0)	1021 (83.4)	210 (75.5)	0.007
One unreactive	100 (6.7)	77 (6.3)	23 (8.3)	
Both unreactive	171 (11.4)	126 (10.3)	45 (16.2)	
Glasgow coma scale, median (IQR)	9.00 [4.00, 14.00]	10.00 [4.00, 14.00]	6.00 [3.00, 12.00]	< 0.001
Hb before transfusion, median (IQR)	–	–	8.4 (7.7–8.5)	
More than one transfusion, n (%)	–	–	165 (56.5)	
GCS motor score, n (%)				
1	459 (29.5)	357 (28.1)	102 (35.7)	< 0.001
2	57 (3.7)	45 (3.5)	12 (4.2)	
3	69 (4.4)	50 (3.9)	19 (6.6)	
4	119 (7.6)	89 (7.0)	30 (10.5)	
5 or 6	852 (54.8)	729 (57.4)	123 (43.0)	
Prehospital or ED Hypoxia, n (%)	201 (13.6)	145 (12.0)	56 (20.7)	< 0.001
Prehospital or ED Hypotension, n (%)	210 (14.1)	131 (10.8)	79 (28.5)	< 0.001
ICP monitoring, n (%)	742 (46.8)	551 (42.5)	191 (65.4)	< 0.001
Cause of injury, n (%)				< 0.001
Incidental fall	629 (41.1)	548 (43.9)	81 (28.4)	
Other	121 (7.9)	102 (8.2)	19 (6.7)	
Road traffic accident	669 (43.7)	506 (40.6)	163 (57.2)	
Suicide attempt	37 (2.4)	25 (2.0)	12 (4.2)	
Violence/assault	76 (5.0)	66 (5.3)	10 (3.5)	
Marshall CT score				
1	136 (9.9)	119 (10.4)	17 (7.2)	0.070
2	663 (48.2)	564 (49.5)	99 (41.9)	
3	108 (7.9)	85 (7.5)	23 (9.7)	
4	18 (1.3)	14 (1.2)	4 (1.7)	
5	1 (0.1)	1 (0.1)	0 (0.0)	
6	449 (32.7)	356 (31.3)	93 (39.4)	
Mortality at six months, n (%)				
Overall	273 (20.0)	209 (19.1)	64 (23.7)	0.110
Weekly Hb < 7.5 g/dL	37 (29.4)			
Weekly Hb between 7.5 and 9.5 g/dL	128 (23.7)			
Weekly Hb > 9.5 g/dL	108 (15.5)			
GOSE < 5 at six Months, n (%)				
Overall	598 (43.9)	445 (40.7)	153 (56.7)	< 0.001
Weekly Hb < 7.5 g/dL	71 (56.3)			
Weekly Hb between 7.5 and 9.5 g/dL	296 (54.9)			
Weekly Hb > 9.5 g/dL	231 (33.1)			

Patients are categorized into two groups according to RBC transfusion (at least one during the first week of ICU admission)

Hb: Haemoglobin; GCS: Glasgow coma scale; GOSE: Glasgow outcome scale extended; ISS: injury severity score; AIS: abbreviated injury scale; ED: emergency department; ICP: intracranial pressure; CT: computed tomography; SD: standard deviation. Any major extracranial injury AIS ≥ 3 defines all the patients with at least one major extracranial injury in any AIS anatomical region

### Haemoglobin values within 48 h from admission and outcomes

**Fig. 2:**
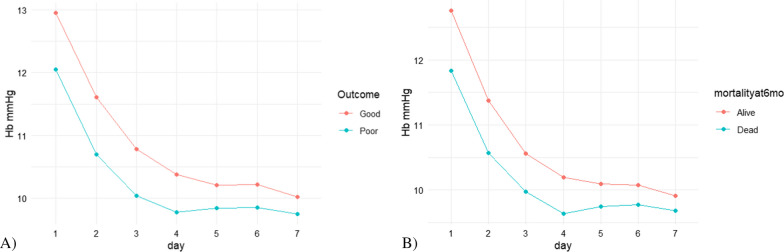
Haemoglobin values over time according to outcomes. Values of Hb over time according to unfavourable outcomes at 6 months (**A**) and mortality at 6 months (**B**). Patients with lower haemoglobin values on admission and during the first week of intensive care unit had a higher occurrence of unfavourable neurological outcomes (identified as GOSE < 5) and mortality at 6 months

**Fig. 3:**
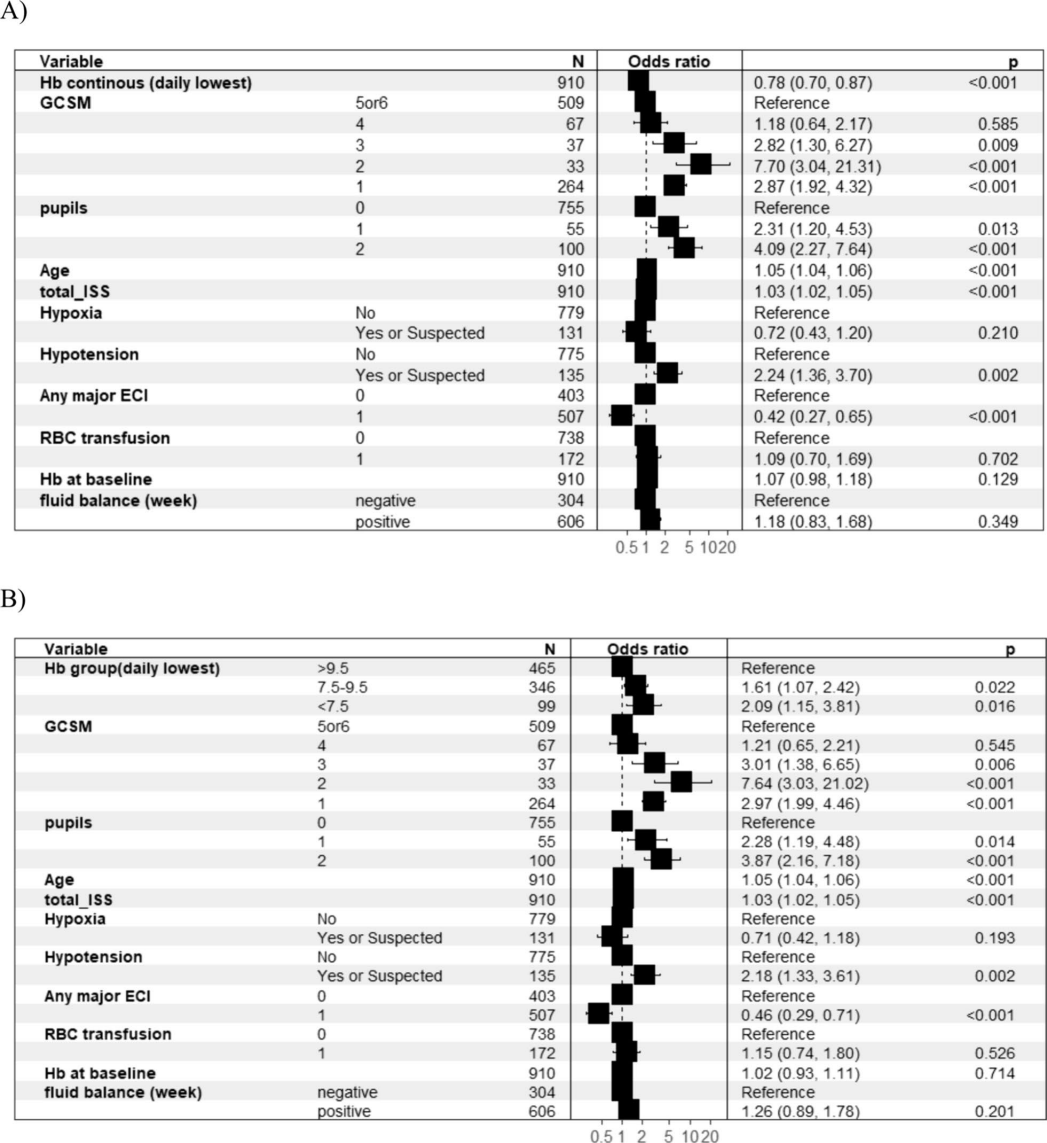
Haemoglobin values and unfavourable outcome. Results of logistic models on unfavourable outcomes at six months (GOSE < 5) and **A** the daily minimum value of haemoglobin during the first week of ICU stay (continuous value); **B** between haemoglobin subgroups (< 7.5 g/dL, 7.5–9.5 g/dL, and > 9.5 g/dL) during the first week of ICU stay. Any ECI; Any major extracranial injury AIS ≥ 3 defines all the patients with at least one major extracranial injury in any AIS anatomical region

598 (43.9%) patients experienced an unfavourable neurological outcome at six months. Anaemic patients during the first week of ICU admission had a higher occurrence of unfavourable neurological outcomes (Table 2, Fig. 2A). In a multivariable logistic regression analysis, adjusted for confounders (age, ISS, presence of prehospital hypotension or hypoxia, presence of any extracranial injury, GCS motor score, haemoglobin on admission, need of transfusion in the first week and weekly fluid balance), the increase of haemoglobin value was independently associated with the decrease of occurrence of unfavourable neurological outcome (OR 0.78; 95% CI 0.70–0.87, Fig. 3A). Both haemoglobin values less than 7.5 g/dL (OR 2.09; 95% CI 1.15–3.81) and between 7.5 and 9.5 g/dL (OR 1.61; 95% CI 1.07–2.42) were independently associated with an increase of unfavourable neurological outcome (Fig. 3B).

**Fig. 4:**
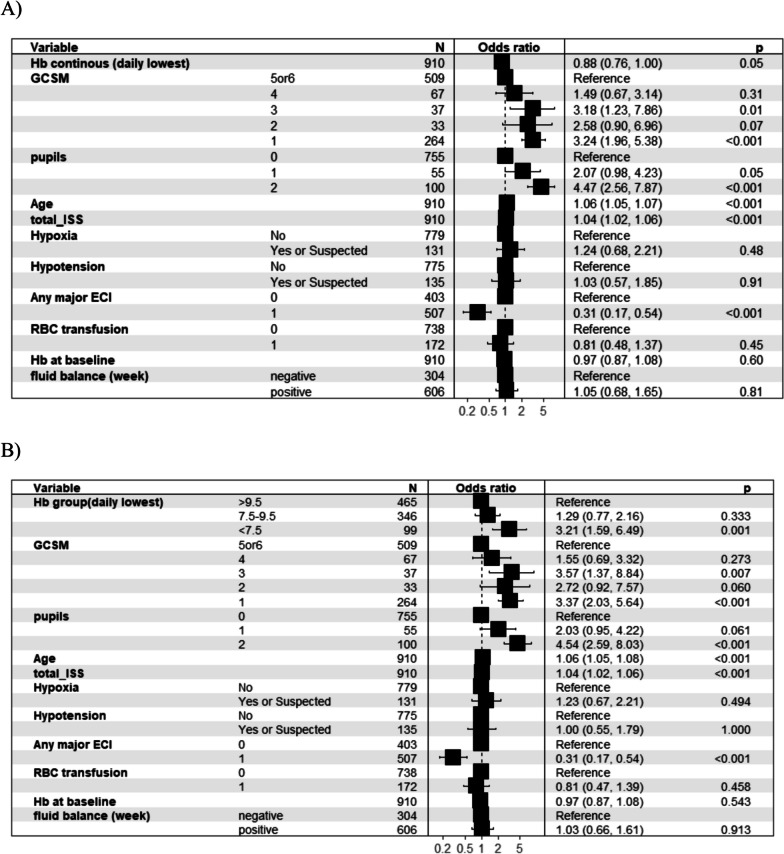
Haemoglobin values and mortality. Results of logistic models on mortality at 6 months and: **A** the daily minimum value of haemoglobin during the first week of ICU stay (continuous value); **B** between haemoglobin subgroups (< 7.5 g/dL, 7.5–9.5 g/dL, and > 9.5 g/dL) during the first week of ICU stay. Any ECI; Any major extracranial injury AIS ≥ 3 defines all the patients with at least one major extracranial injury in any AIS anatomical region

The overall 6-month mortality rate was 20% (n = 273). Anaemic patients during the first week of ICU admission had higher mortality rates than others (Table 2, Fig. 2B). In a multivariable logistic regression analysis, adjusted for confounders (age, ISS, presence of prehospital hypotension or hypoxia, presence of any extracranial injury, GCS motor score, haemoglobin on admission, need of transfusion in the first week and weekly fluid balance), the increase of haemoglobin value was independently associated with a decrease of mortality at 6 months (OR 0.88; 95% CI 0.76–1.00), as shown in Fig. 4A; specifically, haemoglobin values less than 7.5 g/dL (OR 3.21; 95% CI 1.59–6.49) during the first seven days of ICU stay was independently associated with an increase of mortality (Fig. 4B). No association was found with haemoglobin values between 7.5 and 9.5 g/dL (OR 1.29; 95% CI 0.77–2.16).

Consistent results were found in the sensitivity analysis including isolated TBI patients (Supplemental Figs. 5 and 6).

## Discussion

Our study observed a relatively low prevalence of anaemia among TBI patients admitted to ICU across various European centers. However, patients with lower haemoglobin values showed a higher incidence of unfavourable neurological outcomes at 6 months and of mortality. Only a minority of TBI patients required RBC transfusions, with significant variability in transfusion strategies among centers.

Anaemia is a multifactorial condition observed after head trauma [21]. However, the prevalence of anaemia remains challenging to assess. Several studies defined anaemia in TBI patients using a different haemoglobin threshold (e.g. < 10.0–12.0 g/dL) as the one we used [22–24]. Normovolemic anaemia typically triggers compensatory mechanisms, such as increasing oxygen extraction fraction and delivery, by elevating cardiac output and cerebral blood flow [25, 26]. However, this physiological response may falter in the injured brain due to impaired autoregulation, resulting in reduced cerebral oxygen delivery for higher haemoglobin values than healthy subjects, predisposing to secondary brain injury [6, 27].

Additionally, haemorrhagic shock [28], a frequent occurrence in polytrauma patients, can induce cerebral ischemia by reducing cerebral perfusion pressure secondary to systemic hypotension [29]. In this study, we did not explicitly investigate mechanisms beyond the onset of anaemia. At the same time, it is plausible that the mechanisms and timing of anaemia occurrence could influence its association with adverse outcomes. Our cohort's low anaemia prevalence reflects the change in the TBI population. With the ageing of the population in high-income countries, falls now represent a much more significant cause of TBI as opposed to multiple traumas becoming less frequent [11].

Consistent with our findings, several prior investigations have also reported the association of anaemia with adverse outcomes following TBI. In a retrospective analysis involving 1150 TBI patients, mortality significantly increased with haemoglobin concentrations below 9.0 g/dL ﻿[11]. Similarly, another retrospective study of 939 TBI patients identified initial and lowest haemoglobin values as significant predictors of unfavourable outcomes, with a 33% increase in the likelihood of a favourable outcome for every 1.0 g/dL rise in haemoglobin concentration ﻿[5]. Finally, an independent association between an average haemoglobin concentration below 9.0 g/dL over 7 days and heightened hospital mortality has also been reported ﻿[31]. Notably, the effects of reduced haemoglobin values on functional outcome occurred for values below 9.5 g/dL, resulting in a higher trigger for transfusion than commonly used (e.g. restrictive strategy) in other critically ill patients [19].

Our study was, however, designed to evaluate the association of haemoglobin values with long-term outcomes, not the effectiveness of RBC transfusions in improving outcomes. Whether aiming for a higher haemoglobin value improves outcomes in TBI patients remains uncertain. Although data from observational studies yield inconclusive findings ﻿[24], there appears to be a suggestion that transfusion might elevate the risk of neurological complications, potentially exacerbating neurological outcomes and increasing mortality [32]. However, this observation could merely reflect the heightened clinical severity of patients necessitating transfusions. Moreover, a meta-analysis encompassing 23 studies involving TBI patients found no difference in mortality between those who underwent RBC transfusion and those who did not [33].

Nevertheless, the analysis identified considerable heterogeneity in the threshold utilised to trigger RBC transfusion, which could have confounded the findings. Interestingly, our study revealed significant variability among centers and countries regarding transfusion practices in TBI patients, consistent with prior research ﻿[24, 34]. An international survey involving 868 ICU physicians treating acute brain injury found that 54% would administer an RBC transfusion when the haemoglobin concentration was 7.0–8.0 g/dL. However, half of these intensivists indicated they would employ a higher threshold for traumatic brain injury, subarachnoid haemorrhage, or ischemic stroke cases [35]. Another survey of 78 intensivists and neurosurgeons from 66 centers reported that 41% of respondents used a transfusion protocol with a threshold haemoglobin concentration of 7.0–9.0 g/dL for TBI patients. In contrast, 59% opted for a threshold above 9 g/dL [36]. These findings were corroborated by an international survey conducted in Canada, the United States of America, and the UK ﻿[37].

This high variability observed in our study may be attributed to the dearth of high-quality evidence guiding practice and the development of transfusion guidelines specific to acute brain injury, particularly TBI. Only a few studies, all characterised by low-quality evidence, have compared the outcomes of different transfusion thresholds in TBI patient populations. For instance, a subgroup analysis of 67 TBI patients from the multicentr randomised Transfusion Requirements in Critical Care (TRICC) trial revealed no differences in 30-day mortality rates, hospital lengths of stay, or the development of multiple organ dysfunction between groups [18]. Similarly, a retrospective comparison of 1565 TBI patients managed with restrictive or liberal transfusion strategies found no significant differences in clinical outcomes [38]. These results were further supported by a 200 patients’ trial in which patients were randomised to a liberal or restrictive strategy and found no significant differences in the proportion of patients with 6-month favourable outcomes [9]. However, a substantial proportion of patients in the liberal group never received a transfusion since non-anaemic patients were considered at enrolment in this trial. On the other hand, a feasibility study involving 44 patients with moderate or severe TBI showed that patients randomised to a liberal transfusion strategy had lower hospital mortality, with a clear trend towards more favourable neurological outcomes at six months [20]. Two large-scale ongoing randomised trials [23, 39] have examined two different haemoglobin thresholds to initiate RBC transfusions in this setting and will provide important information for managing anaemic TBI patients.

This study has several limitations. Firstly, being an observational study, our results are hypothesis-generating rather than conclusive. Secondly, various studies examining transfusion practices have employed different haemoglobin cut-offs to define restrictive and liberal strategies. The different cut-offs utilised may be arbitrary, complicating comparisons with other studies. Thirdly, our data originate from European databases in high-income countries. Thus, the generalizability of our results to different populations and middle to low-income countries may be limited. Fourthly, our inclusion of TBIs within the context of polytrauma could have influenced our results, as polytrauma patients often receive red blood cell transfusions and undergo additional procedures as part of initial resuscitation in the setting of haemorrhagic shock.

## Conclusions

In this study, anaemia was significantly associated with unfavourable neurological outcomes and mortality rates in TBI patients requiring ICU admission. Transfusion policy was heterogeneous among centers.

### Supplementary Information

Supplementary Material 1.

Supplementary Material 2.

## Data Availability

The data supporting the study findings are available upon reasonable request after approval of a proposal from the corresponding author (GC). Data collected for the analysis will be made available to others, including deidentified individual participant data and a data dictionary defining each field in the set. Related documents, such as the study protocol, statistical analysis plan, and informed consent form, will also be available.

## Abbreviations

AIS: Abbreviated injury scale
CI: Confidence interval
ESM: Electronic Supplementary Material
GCS: Glasgow coma scale
GOSE: Glasgow outcome scale extended
Hb: Haemoglobin
ICU: Intensive care unit
IQR: Interquartile range
ISS: Injury severity score
OR: Odds ratio
RBC: Red blood cell
RCT: Randomized clinical trial
SD: Standard deviation
STROBE: Strengthening the Reporting of Observational Studies in Epidemiology guidelines
TBI: Traumatic brain injury
TRALI: Transfusion-related acute lung injury
TRICC: Transfusion Requirements in Critical Care Trial
