# Supplementary material for: Haemoglobin values, transfusion practices, and long-term outcomes in critically ill patients with traumatic brain injury: a secondary analysis of CENTER-TBI
Source: Crit Care. 2024 Jun 14;28:199. doi: 10.1186/s13054-024-04980-6 (PMC11177426; doi:10.1186/s13054-024-04980-6)
Supplement: Supplementary file 1 — Supplementary Material 1. [file 13054_2024_4980_MOESM1_ESM.docx]

**Electronic supplementary material**

[“Strengthening the Reporting of Observational Studies in Epidemiology (STROBE)” statement guidelines. 2](#_Toc162261292)

[Figure S1. Flowchart for patients’ inclusion in the sub-analysis 10](#_Toc162261293)

[Table S1. Variation of haemoglobin levels of ICU and fluid balance during the first week. 10](#_Toc162261294)

[Figure S2. Variation of haemoglobin levels during the first week of ICU. 11](#_Toc162261295)

[Table S2. Variability of haemoglobin levels at baseline between countries. 11](#_Toc162261296)

[Figure S3. Delta values of haemoglobin (median negative reduction of 2.7 g/dL) between day 7 and day 1 across countries (A) and centres (B) 12](#_Toc162261297)

[Figure S4. Values of Hb before transfusion in each country 13](#_Toc162261298)

[Figure S5. Transfusion practices amongst countries. 14](#_Toc162261299)

[Abbreviations of country: 14](#_Toc162261300)

# “Strengthening the Reporting of Observational Studies in Epidemiology (STROBE)” statement guidelines.

|  | **Item No.** | **Recommendation** | **Page  No.** | **Relevant text from manuscript** |
| --- | --- | --- | --- | --- |
| **Title and abstract** | 1 | (*a*) Indicate the study’s design with a commonly used term in the title or the abstract | 1 | Haemoglobin levels, transfusions and outcomes after traumatic brain injury: Insight from Center-TBI |
|  |  | (*b*) Provide in the abstract an informative and balanced summary of what was done and what was found | 1 |  |
| **Introduction** | | | |  |
| Background/rationale | 2 | Explain the scientific background and rationale for the investigation being reported | 2-3 | Traumatic brain injury (TBI) encompasses a spectrum of injuries resulting from external mechanical forces to the brain, leading to varying degrees of neurological impairment. While mild cases may present with transient symptoms, severe TBI can have profound and lasting consequences, including cognitive deficits, motor impairments, and emotional disturbances.  Central to the management of TBI is the prevention and mitigation of secondary brain injuries, which can occur in the hours to days following the initial trauma. Secondary insults, such as hypoxia, hypotension, cerebral oedema and ischemia, can exacerbate primary brain damage and significantly impact patient outcomes. Among those, anaemia frequently coexists with TBI and has emerged as a critical determinant of patient outcomes. The pathophysiology of anaemia in TBI is multifactorial, with contributions from acute blood loss, haemodilution, coagulopathy, and impaired erythropoiesis. Furthermore, anaemia in TBI is associated with a cascade of deleterious effects, including cerebral hypoxia, compromised tissue perfusion and increased susceptibility to secondary brain injuries. Consequently, patients with TBI and concomitant anaemia often experience worse neurological outcomes, prolonged hospitalisations, and increased mortality rates compared to non-anaemic TBI patients.  The management of anaemia in TBI patients often involves administering red blood cells (RBC) transfusions to improve oxygen delivery and mitigate the adverse effects of low haemoglobin levels. However, the use of RBC transfusions in this population is fraught with controversy due to concerns regarding potential complications, such as transfusion-related acute lung injury (TRALI) and increased risk of infections. Moreover, emerging evidence suggests that exposure to RBC transfusion may be associated with worse outcomes in TBI patients. Thus, clinicians face the challenge of balancing the potential benefits and risks of anaemia and RBC transfusions in this context, necessitating a nuanced approach tailored to individual patient factors and clinical contexts.  Several knowledge gaps and inconsistencies persist without well-conducted randomised trials in this field. Firstly, the association between anaemia and poor outcomes in TBI has been frequently evaluated in single center studies, with significant bias on results due to local practice and the limited correction for potential confounders. Moreover, considerable heterogeneity exists in transfusion practices across different healthcare settings, with variations in haemoglobin thresholds, triggers, and strategies. Consequently, there is a pressing need for large, multi-centric studies to elucidate the potential role of anaemia as a predictive factor for poor outcomes after TBI and to refine how transfusion algorithms impact these findings. |
| Objectives | 3 | State specific objectives, including any prespecified hypotheses | 3 | This article aimed to explore Hb levels after TBI, RBC transfusion practices and their association with outcomes in a large European cohort of TBI patients. |
| Methods | | | |  |
| Study design | 4 | Present key elements of study design early in the paper | 3 | The CENTER-TBI study (clinicaltrials.gov NCT02210221) is a longitudinal, prospective collection of data from TBI patients across 65 centers in Europe and Israel. Details regarding the study design, methodology, screening and enrolment process have been previously described. The study was approved by the Medical Ethics Committees of each participating center, and informed consent was obtained according to local regulations (https://www.center-tbi.eu/project/ethical-approval). This substudy, which was pre-registered on the CENTER-TBI proposal platform, was approved by the Steering Committee and by the CENTER-TBI proposal review committee. |
| Setting | 5 | Describe the setting, locations, and relevant dates, including periods of recruitment, exposure, follow-up, and data collection | 2-3 | Data were de identified and stored on a secure database,hosted by the International Neuroinformatics Coordinating Facility in Stockholm, Sweden. Details on data collection and management of the CENTER-TBI study have been previously published. Collected data encompassed various patient parameters, including demographic characteristics, pre-existing comorbidities, mechanism of TBI and admission assessments, neurological status (e.g., Glasgow Coma Scale - GCS, pupillary reactivity), and presence of extracranial injuries (quantified by the total Injury Severity Score - ISS, with major extracranial injury defined by an Abbreviated Injury Scale - AIS score ≥ 3).  Additionally, variables pertaining to the requirement for neurosurgical interventions and intracranial pressure (ICP) monitoring, specifics of ICP management therapies (e.g. fluids balance) , necessity for extracranial and intracranial surgeries (e.g., damage control procedures), need for blood transfusions , and utilisation of intubation, mechanical ventilation, and tracheostomy were documented. Follow up was obtained at 6 months. |
| Participants | 6 | (a) Cohort study—Give the eligibility criteria, and the sources and methods of selection of participants. Describe methods of follow-up | 3-4 | Inclusion criteria for this analysis were all patients included in the CENTER-TBI study aged ≥ 18 years old, who required intensive care unit (ICU) admission and with data available on haemoglobin levels and red blood cell transfusions, as well as data on six-month mortality and neurological outcome (Glasgow Outcome Score – Extended, GOSE). Within the CENTER-TBI cohort, TBI patients eligible for inclusion in this study met the following criteria:  a) aged 18 years or older;  b) admitted to the intensive care unit (ICU);  c) had at least one haemoglobin levels available measured at baseline (e.g. within 48h from hospital admission) and at least twice during the first week after admission; d) underwent assessment of 6-month neurological outcomes using the Glasgow Outcome Score-Extended (GOSE).. When several haemoglobin measurements were reported daily, the lowest measurement was considered for the analysis. |
|  |  |  |  |  |
| Variables | 7 | Clearly define all outcomes, exposures, predictors, potential confounders, and effect modifiers. Give diagnostic criteria, if applicable | 4 | Data collected included patients’ demographic characteristics, pre-injury comorbidities, TBI mechanism, neuroradiological features (as for Marshall CT score), neurological status at admission (as for Glasgow Coma Scale (GCS)), need for neurosurgical treatment and intracranial pressure (ICP) monitoring, treatment used for ICP management (i.e. therapy intensity level (TIL), etc.), presence of extracranial injury (defined as an Abbreviated Injury Scale (AIS) ≥ 3 and the total Injury Severity Score (ISS)), and need for extracranial and cranial surgeries (e.g. damage control procedures, etc.), need for blood transfusion, need for intubation and mechanical ventilation, need for tracheotomy, arterial blood gas (ABG) values (i.e. pH, partial pressure of oxygen (PaO2), partial pressure of carbon dioxide (PaCO2), base excess), and laboratory data (e.g. creatinine, glucose) at hospital arrival and clinical outcomes.  Our primary outcome was the Glasgow outcome scale extended (GOSE), an 8-point ordinal scale, measured at six months [13]. We defined an unfavourable neurological outcome as a GOSE <5. Our secondary outcomes were mortality and the proportion of transfusions across centers. |
| Data sources/ measurement | 8* | For each variable of interest, give sources of data and details of methods of assessment (measurement). Describe comparability of assessment methods if there is more than one group | 4 | Data collection and management procedures for the CENTER-TBI study have been previously outlined [11,12]. The CENTER-TBI core database v3.0 was accessed and retrieved via the Opal data warehouse [14]. Collected data encompassed various patient parameters, including demographic characteristics, pre-existing comorbidities, mechanism of TBI and admission assessments such as neuroradiological findings [15], neurological status (e.g., Glasgow Coma Scale - GCS, pupillary reactivity), and presence of extracranial injuries (quantified by the total Injury Severity Score - ISS, with major extracranial injury defined by an Abbreviated Injury Scale - AIS score ≥ 3) [16]. Additionally, variables pertaining to the requirement for neurosurgical interventions and intracranial pressure (ICP) monitoring, specifics of ICP management therapies (e.g. fluids balance) [17], and the necessity for extracranial and intracranial surgeries (e.g., damage control procedures). |
| Bias | 9 | Describe any efforts to address potential sources of bias |  |  |
| Study size | 10 | Explain how the study size was arrived at | 4 | From a total of 2006 ICU adult patients enrolled in CENTER-TBI, 1590 met eligibility criteria and were included in our study. Of whom, 1231 had haemoglobin values available on admission. |

| Quantitative variables | 11 | Explain how quantitative variables were handled in the analyses. If applicable, describe which groupings were chosen and why | 5 | Continuous variables were summarised using medians and quartiles and categorical variables using frequencies and percentage.  Delta Hb defined as the difference between the Hb values at day 1 and day 7.  Regarding the classification of "liberal" or "restrictive" among countries in transfusion, both continuous Hb values before transfusion and the proportion of two types of policy were calculated. Only centers with at least ten or more RBC transfusions were deemed suitable for transfusion-related analyses. Centers or countries with less than 10 enrolled patients were allocated in a group "Other" |
| --- | --- | --- | --- | --- |
| Statistical methods | 12 | (a) Describe all statistical methods, including those used to control for confounding | 5 | Continuous variables were summarised using medians and quartiles and categorical variables using frequencies and percentage. Comparison between groups were performed using the Wilcoxon rank-sum test, chi-squared or the Fisher exact test as appropriate.  To evaluate the centres and countries variabilities, a linear mixed model was used using delta Hb defined as the difference between the Hb values at day 1 and day 7 as outcome and adjusted for case -mix (demographics and clinical variables, e.g. age, ISS, GCS motor score and baseline pupil abnormality) and centers/countries as random effect. The variability was shown as caterpillar plot. To test if variability is significant different, Anova test was used to comparing models with and without the random effect.  The primary outcome analysis was adjusted using the logistic regression model analysis on unfavourable outcome at 6 months (GOSE <5) and mortality at 6 months, in which variables with a significant difference (p<0.05) in the univariate analysis and clinical relevance were included. The results were reported as odds ratio (OR) and corresponding 95% confidence interval (CI). A type I error rate of 0.05 was employed. All analyses were conducted using R software (version 4.0.3). |
|  |  | (b) Describe any methods used to examine subgroups and interactions | 5 |  |
|  |  | (c) Explain how missing data were addressed |  |  |
|  |  |  |  |  |
|  |  | (d) Describe any sensitivity analyses |  |  |
| Results | | | | |
| Participants | 13* | (a) Report numbers of individuals at each stage of study—eg numbers potentially eligible, examined for eligibility, confirmed eligible, included in the study, completing follow-up, and analyzed |  | ESM, FIG S1 |
|  |  | (b) Give reasons for non-participation at each stage |  | ESM, FIG S1 |
|  |  | (c) Consider use of a flow diagram |  | ESM, FIG S1 |
| Descriptive data | 14* | (a) Give characteristics of study participants (eg demographic, clinical, social) and information on exposures and potential confounders | 16 | TABLE 1 |
|  |  | (b) Indicate number of participants with missing data for each variable of interest |  | ESM, FIG S1 |
|  |  | (c) Cohort study—Summaries follow-up time (eg, average and total amount) |  | ESM, FIG S1 |
| Outcome data | 15* | Cohort study—Report numbers of outcome events or summary measures over time | 17 | FIG3A-B |
|  |  | Case-control study—Report numbers in each exposure category, or summary measures of exposure |  |  |
|  |  | Cross-sectional study—Report numbers of outcome events or summary measures |  |  |
| Main results | 16 | (a) Give unadjusted estimates and, if applicable, confounder-adjusted estimates and their precision (eg, 95% confidence interval). Make clear which confounders were adjusted for and why they were included | 16-20 | Population characteristics and Hb values at baseline: TABLE 1.  Haemoglobin values at baselined over the week: FIG1, TABLES1, FIGS1  Haemoglobin and center/countries variability: FIGS3a and FIGS3b  Transfusion Practices: TAB2, FIGS4  Haemoglobin values and outcome: FIG2, FIG3a-3b |
|  |  | (b) Report category boundaries when continuous variables were categorized |  |  |
|  |  | (c) If relevant, consider translating estimates of relative risk into absolute risk for a meaningful time period |  |  |
| Other analyses | 17 | Report other analyses done—eg analyses of subgroups and interactions, and sensitivity analyses | 16-20 | Haemoglobin values and outcome:TAB1, FIG2, FIG3a-3b |
| Discussion |  |  |  |  |
| Key results | 18 | Summaries key results with reference to study objectives | 6-7 | In this study, anaemia was significantly associated with poor neurological outcomes and mortality rates in TBI patients requiring ICU admission. Transfusion policy was heterogeneous among centers. |
| Limitations | 19 | Discuss limitations of the study, taking into account sources of potential bias or imprecision. Discuss both direction and magnitude of any potential bias | 7 | This study has several limitations. Firstly. being an observational study, our results are hypothesis-generating rather than conclusive. Secondly, various studies examining transfusion practices have employed different haemoglobin cut-offs to define restrictive and liberal strategies. The different cut-offs we utilised may be arbitrary, complicating comparisons with other studies. Thirdly, our data originate from European databases in high-income countries, thus the generalizability of our results to other populations and to middle to low-income countries may be limited. Fourthly, our inclusion of TBIs within the context of polytrauma could have influenced our results, as polytrauma patients often receive red blood cell transfusions as part of initial resuscitation in the setting of haemorrhagic shock. |
| Interpretation | 20 | Give a cautious overall interpretation of results considering objectives, limitations, multiplicity of analyses, results from similar studies, and other relevant evidence | 7 | This study observed a relatively low prevalence of anaemia among TBI patients admitted to ICU across various European centers. However, patients with lower haemoglobin levels during the first week of ICU exhibited elevated mortality rates and a higher incidence of poor neurological outcomes at 6 months than others. Only a minority of TBI patients required red blood cell transfusions, with a significant variability in transfusion strategies among centers, even within the same country. |
| Generalizability | 21 | Discuss the generalizability (external validity) of the study results |  |  |
| Other information |  |  |  |  |
| Funding | 22 | Give the source of funding and the role of the funders for the present study and, if applicable, for the original study on which the present article is based. |  |  |

*Give information separately for cases and controls in case-control studies and, if applicable, for exposed and unexposed groups in cohort and cross-sectional studies.

**Note:** An Explanation and Elaboration article discusses each checklist item and gives methodological background and published examples of transparent reporting. The STROBE checklist is best used in conjunction with this article (freely available on the Web sites of PLoS Medicine at http://www.plosmedicine.org/, Annals of Internal Medicine at http://www.annals.org/, and Epidemiology at http://www.epidem.com/). Information on the STROBE Initiative is available at www.strobe-statement.org

Figure S1. Flowchart for patients’ inclusion in the sub-analysis.


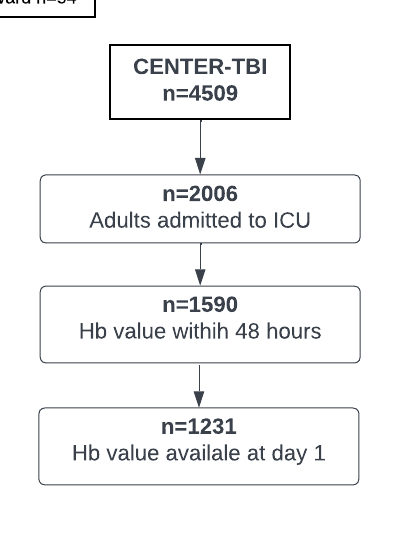


Abbreviations: ICU; intensive care unit; Hb; haemoglobin;

Table S1. Variation of haemoglobin levels of ICU and fluid balance during the first week.

|  |  | **days** | | | | | | |  |  |
| --- | --- | --- | --- | --- | --- | --- | --- | --- | --- | --- |
|  |  | **1** | **2** | **3** | **4** | **5** | **6** | **7** | **Overall** | **Fluid balance (litres)** |
| **N. of patients** | **Groups** | **1231** | **1400** | **1253** | **1117** | **989** | **814** | **605** | **1590** | **Mean(SD)**  **1.6(3.6)** |
| **Cut-off (%)** | **<7.5 g/dL** | 15 (1.2) | 19 (1.4) | 40 (3.2) | 58 (5.2) | 35 (3.5) | 40 (4.9) | 24 (4.0) | 138 (8.7) | 3.3 (4.2) |
|  | **7.5-9.5 g/dL** | 106 (8.6) | 241 (17.2) | 375 (29.9) | 383 (34.3) | 369 (37.3) | 285 (35.0) | 236 (39.0) | 624 (39.2) | 2.2 (4.1) |
|  | **>9.5 g/dL** | 1110 (90.2) | 1140 (81.4) | 838 (66.9) | 676 (60.5) | 585 (59.2) | 489 (60.1) | 345 (57.0) | 828 (52.1) | 0.9 (2.8) |

Figure S2. Variation of haemoglobin levels during the first week of ICU.


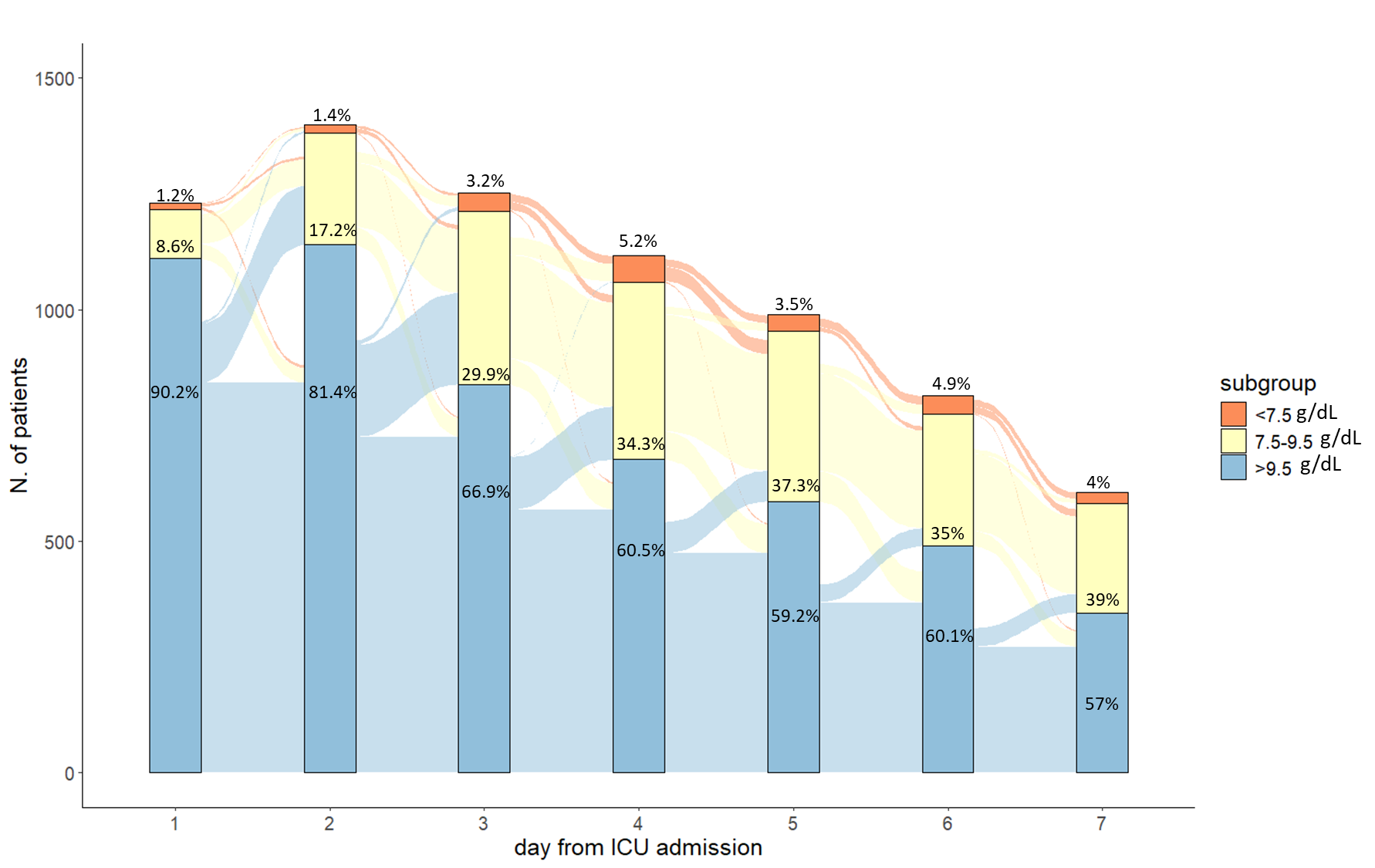


Table S2. Variability of haemoglobin levels at day 1 between countries.

|  | **N** | **Mean (SD)** |
| --- | --- | --- |
| **Overall** | **1231** | **12.6 (2.2)** |
| Country |  |  |
| AT | 91 | 13.3 (1.9) |
| BE | 101 | 12.9 (2.1) |
| DE | 63 | 12.1 (2.3) |
| DK | 2 | 14.6 (0.1) |
| ES | 165 | 12.8 (2.3) |
| FI | 100 | 12.7 (1.8) |
| FR | 71 | 12.7 (2.3) |
| HU | 19 | 13.0 (1.9) |
| IL | 1 | 14.5 (NA) |
| IT | 255 | 12.1 (2.3) |
| LT | 19 | 12.4 (2.2) |
| LV | 7 | 12.9 (1.8) |
| NO | 102 | 13.1 (2.0) |
| RS | 7 | 12.7 (2.7) |
| SE | 67 | 12.1 (2.0) |
| UK | 161 | 12.7 (2.0) |

Abbreviations: SD; standard deviation.

Figure S3. Delta values of haemoglobin (median negative reduction of 2.7 g/dL) between day 7 and day 1 across countries (A) and centres (B). Centers and countries variability was evaluated through linear mixed model (adjusted for age, Total ISS, GCSMotor, Pupils Baseline Derived). This plot shows the deviation from the mean population Hb (mean equal to -2.6) for each family (center/country), together with standard errors. Centers/countries were below or above the population mean but not significantly (for centers A, p=0.24 and for countries B, p=0.09).


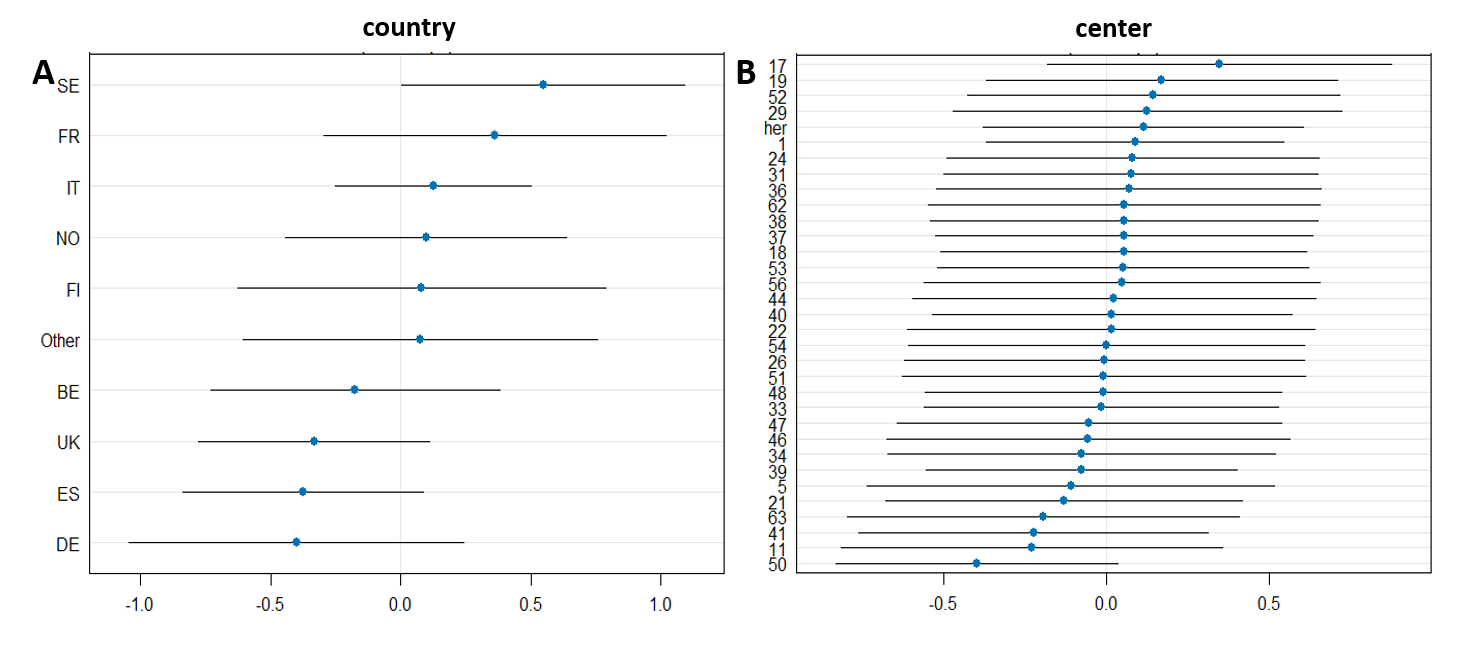
Abbreviations: GCS; Glasgow coma scale; ISS; injury severity score.

Figure S4. Values of Hb before transfusion in each country. Distribution of the 562 transfusion events, divided by countries. The two black lines represent the threshold used for RBC transfusion (“restrictive”: 7.5 g/dL and “liberal”: 9.5 g/dL). The empty triangle represents the mean value.


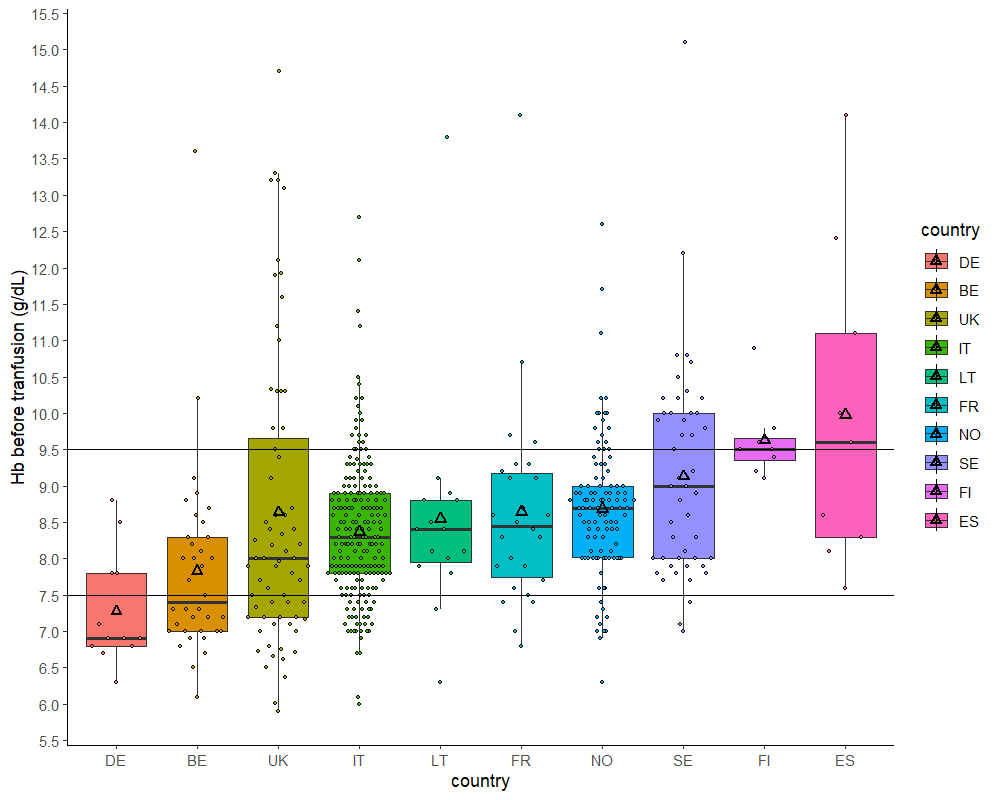


Figure S5. Transfusion practices amongst countries. We defined transfusion practices as "restrictive" or "liberal" based on haemoglobin values before transfusion.


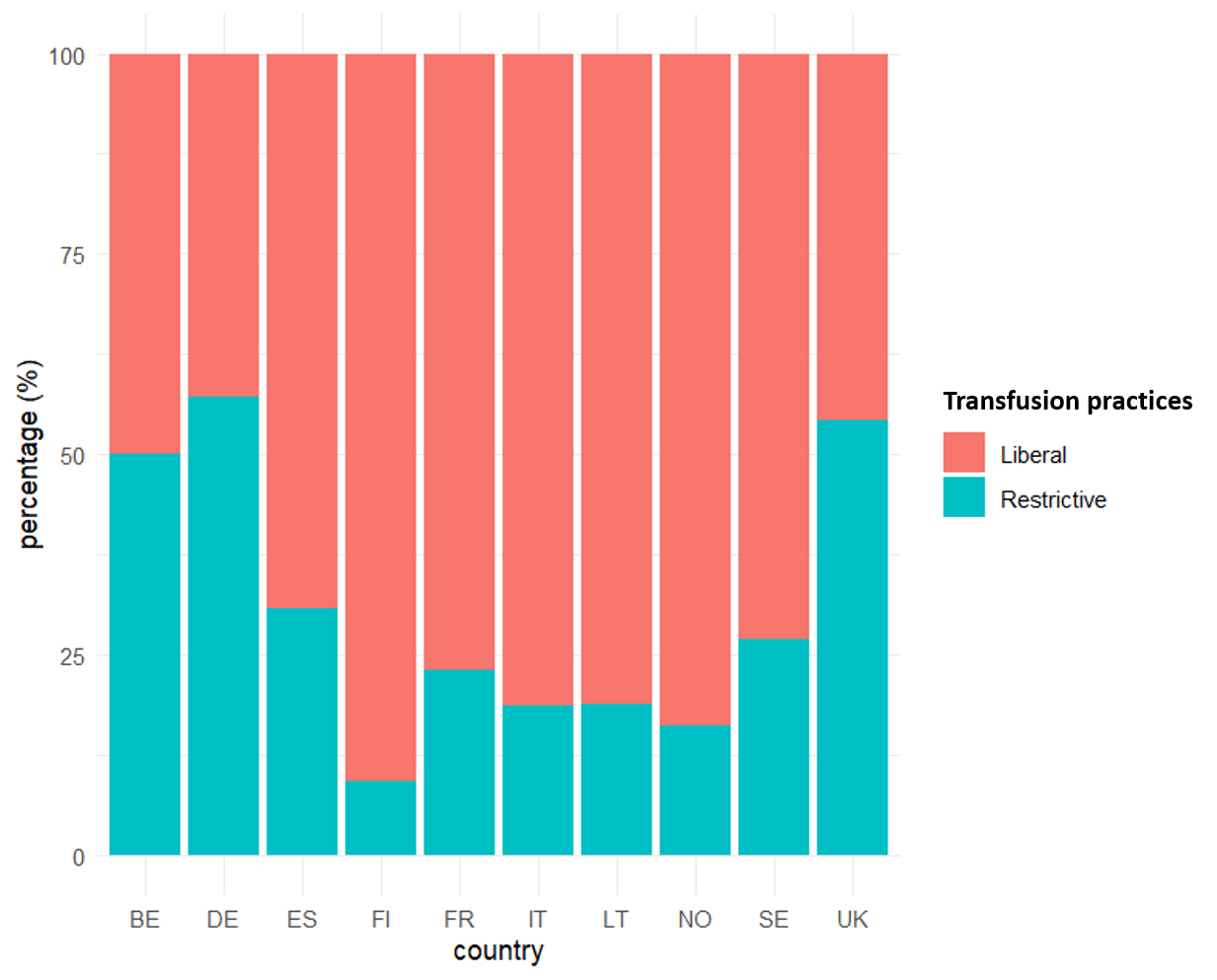


Abbreviations of country

AT, Austria; BE, Belgium; DE, Germany; DK, Denmark; ES, Spain; FI, Finland; FR, France; HU, Hungary; IL, Israel; IT, Italy; LT, Lithuania; LV, Latvia; NO, Norway; RS, Serbia; SE, Sweden; UK, United Kingdom

Figure S5. Sensitivity analysis considering only TBI patients - **Haemoglobin values and unfavourable outcome** - Results of logistic models on unfavourable outcomes at six months (GOSE <5) and A) the daily minimum value of haemoglobin during the first week of ICU stay (continuous value); B) between haemoglobin subgroups (<7.5 g/dL, 7.5-9.5 g/dL, and>9.5 g/dL) during the first week of ICU stay.

Abbreviations: Any ECI; Any major extracranial injury AIS ≥ 3 defines all the patients with at least one major extracranial injury in any AIS anatomical region.

**A)**

**
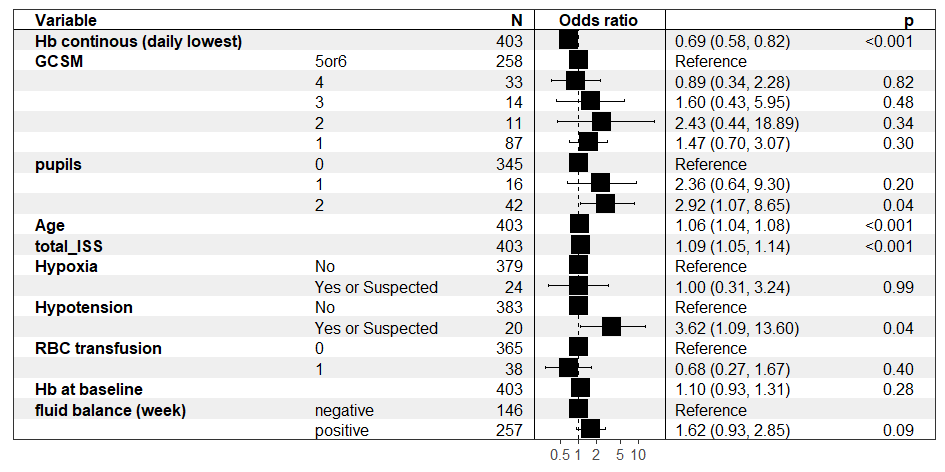
B)**

**
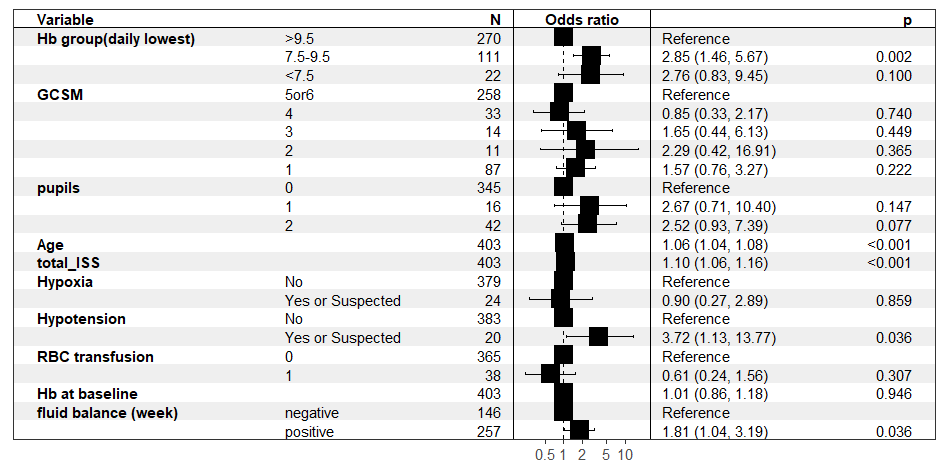
**

Figure S6. Sensitivity analysis considering only TBI patients- **Haemoglobin values and mortality -** Results of logistic models on mortality at six months and: A) the daily minimum value of haemoglobin during the first week of ICU stay (continuous value); B) between haemoglobin subgroups (<7.5 g/dL, 7.5-9.5 g/dL, and>9.5 g/dL) during the first week of ICU stay.

Abbreviations: Any ECI; Any major extracranial injury AIS ≥ 3 defines all the patients with at least one major extracranial injury in any AIS anatomical region.

**A)**

**
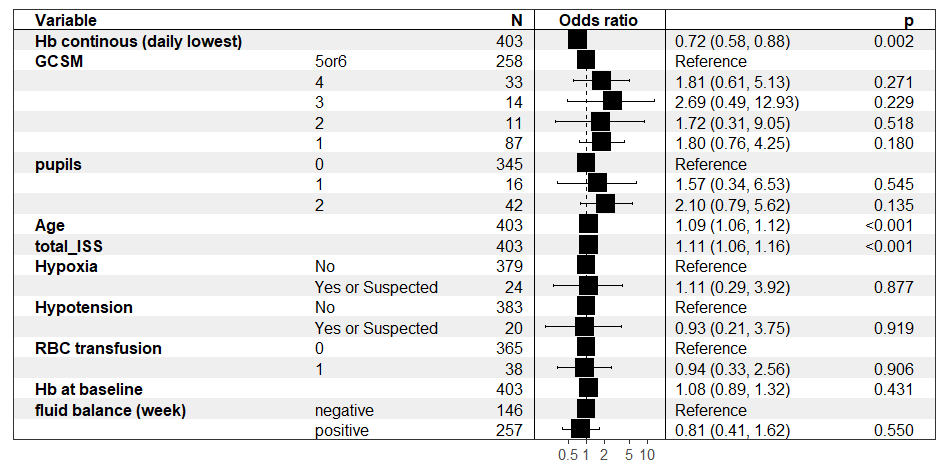
**

**B)**

**
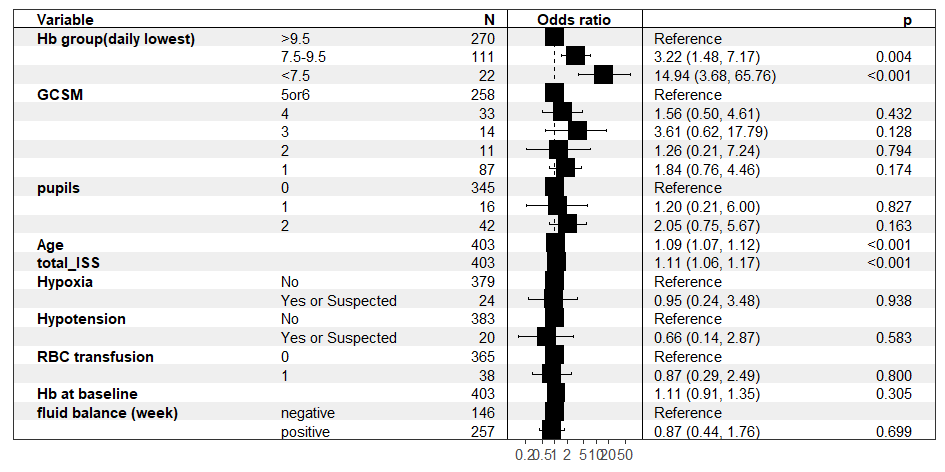
**
