# Supplementary material for: Haemoglobin values, transfusion practices, and long-term outcomes in critically ill patients with traumatic brain injury: a secondary analysis of CENTER-TBI
Source: Crit Care. 2024 Jun 14;28:199. doi: 10.1186/s13054-024-04980-6 (PMC11177426; doi:10.1186/s13054-024-04980-6)
Supplement: Supplementary file 2 — Supplementary Material 2. [file 13054_2024_4980_MOESM2_ESM.docx]

# **The CENTER-TBI participants and investigators**

Cecilia Åkerlund^1^, Krisztina Amrein^2^, Nada Andelic^3^, Lasse Andreassen^4^, Audny Anke^5^, Anna Antoni^6^, Gérard Audibert^7^, Philippe Azouvi^8^, Maria Luisa Azzolini^9^, Ronald Bartels^10^, Pál Barzó^11^, Romuald Beauvais^12^, Ronny Beer^13^, Bo-Michael Bellander^14^, Antonio Belli^15^, Habib Benali^16^, Maurizio Berardino^17^, Luigi Beretta^9^, Morten Blaabjerg^18^, Peter Bragge^19^, Alexandra Brazinova^20^, Vibeke Brinck^21^, Joanne Brooker^22^, Camilla Brorsson^23^, Andras Buki^24^, Monika Bullinger^25^, Manuel Cabeleira^26^, Alessio Caccioppola^27^, Emiliana Calappi ^27^, Maria Rosa Calvi^9^, Peter Cameron^28^, Guillermo Carbayo Lozano^29^, Marco Carbonara^27^, Simona Cavallo^17^, Giorgio Chevallard^30^, Arturo Chieregato^30^, Giuseppe Citerio^31, 32^, Hans Clusmann^33^, Mark Coburn^34^, Jonathan Coles^35^, Jamie D. Cooper^36^, Marta Correia^37^, Amra Čović ^38^, Nicola Curry^39^, Endre Czeiter^24^, Marek Czosnyka^26^, Claire Dahyot‑Fizelier^40^, Paul Dark^41^, Helen Dawes^42^, Véronique De Keyser^43^, Vincent Degos^16^, Francesco Della Corte^44^, Hugo den Boogert^10^, Bart Depreitere^45^, Đula Đilvesi ^46^, Abhishek Dixit^47^, Emma Donoghue^22^, Jens Dreier^48^, Guy‑Loup  Dulière^49^, Ari Ercole^47^, Patrick Esser^42^, Erzsébet Ezer^50^, Martin  Fabricius^51^, Valery L. Feigin^52^, Kelly  Foks^53^, Shirin Frisvold^54^, Alex Furmanov^55^, Pablo Gagliardo^56^, Damien Galanaud^16^, Dashiell Gantner^28^, Guoyi Gao^57^, Pradeep George^58^, Alexandre Ghuysen^59^, Lelde Giga^60^, Ben Glocker^61^, Jagoš Golubovic^46^, Pedro A. Gomez ^62^, Johannes Gratz^63^, Benjamin Gravesteijn^64^, Francesca Grossi^44^, Russell L. Gruen^65^, Deepak Gupta^66^, Juanita A. Haagsma^64^, Iain Haitsma^67^, Raimund Helbok^13^, Eirik Helseth^68^, Lindsay Horton ^69^, Jilske Huijben^64^, Peter J. Hutchinson^70^, Bram Jacobs^71^, Stefan Jankowski^72^, Mike Jarrett^21^, Ji‑yao  Jiang^58^, Faye Johnson^73^, Kelly Jones^52^, Mladen Karan^46^, Angelos G. Kolias^70^, Erwin Kompanje^74^, Daniel Kondziella^51^, Evgenios Kornaropoulos^47^, Lars‑Owe Koskinen^75^, Noémi Kovács^76^, Ana Kowark^77^, Alfonso Lagares^62^, Linda Lanyon^58^, Steven Laureys^78^, Fiona Lecky^79, 80^, Didier Ledoux^78^, Rolf Lefering^81^, Valerie Legrand^82^, Aurelie Lejeune^83^, Leon Levi^84^, Roger Lightfoot^85^, Hester Lingsma^64^, Andrew I.R. Maas^43^, Ana M. Castaño‑León^62^, Marc Maegele^86^, Marek Majdan^20^, Alex Manara^87^, Geoffrey Manley^88^, Costanza Martino^89^, Hugues Maréchal^49^, Julia Mattern^90^, Catherine McMahon^91^, Béla Melegh^92^, David Menon^47^, Tomas Menovsky^43^, Ana Mikolic^64^, Benoit Misset^78^, Visakh Muraleedharan^58^, Lynnette Murray^28^, Ancuta Negru^93^, David Nelson^1^, Virginia Newcombe^47^, Daan Nieboer^64^, József Nyirádi^2^, Otesile Olubukola^79^, Matej Oresic^94^, Fabrizio Ortolano^27^, Aarno Palotie^95, 96, 97^, Paul M. Parizel^98^, Jean‑François Payen^99^, Natascha Perera^12^, Vincent Perlbarg^16^, Paolo Persona^100^, Wilco Peul^101^, Anna Piippo-Karjalainen^102^, Matti Pirinen^95^, Dana Pisica^64^, Horia Ples^93^, Suzanne Polinder^64^, Inigo Pomposo^29^, Jussi P. Posti ^103^, Louis Puybasset^104^, Andreea Radoi ^105^, Arminas Ragauskas^106^, Rahul Raj^102^, Malinka Rambadagalla^107^, Isabel Retel Helmrich^64^, Jonathan Rhodes^108^, Sylvia Richardson^109^, Sophie Richter^47^, Samuli Ripatti^95^, Saulius Rocka^106^, Cecilie Roe^110^, Olav Roise^111,112^, Jonathan Rosand^113^, Jeffrey V. Rosenfeld^114^, Christina Rosenlund^115^, Guy Rosenthal^55^, Rolf Rossaint^77^, Sandra Rossi^100^, Daniel Rueckert^61^ Martin Rusnák^116^, Juan Sahuquillo^105^, Oliver Sakowitz^90, 117^, Renan Sanchez‑Porras^117^, Janos Sandor^118^, Nadine Schäfer^81^, Silke Schmidt^119^, Herbert Schoechl^120^, Guus Schoonman^121^, Rico Frederik Schou^122^, Elisabeth Schwendenwein^6^, Charlie Sewalt^64^, Ranjit D. Singh^101^, Toril Skandsen^123, 124^ , Peter Smielewski^26^, Abayomi Sorinola^125^, Emmanuel Stamatakis^47^, Simon Stanworth^39^, Robert Stevens^126^, William Stewart^127^, Ewout W. Steyerberg^64,^ ^128^, Nino Stocchetti^129^, Nina Sundström^130^, Riikka Takala^131^, Viktória Tamás^125^, Tomas Tamosuitis^132^, Mark Steven Taylor^20^, Aurore Thibaut^78^, Braden Te Ao^52^, Olli Tenovuo^103^, Alice Theadom^52^, Matt Thomas^87^, Dick Tibboel^133^, Marjolein Timmers^74^, Christos Tolias^134^, Tony Trapani^28^, Cristina Maria Tudora^93^, Andreas Unterberg^90^, Peter Vajkoczy ^135^, Shirley Vallance^28^, Egils Valeinis^60^, Zoltán Vámos^50^, Mathieu van der Jagt^136^, Gregory Van der Steen^43^, Joukje van der Naalt^71^, Jeroen T.J.M. van Dijck ^101^, Inge A. M. van Erp^101^, Thomas A. van Essen^101^, Wim Van Hecke^137^, Caroline van Heugten^138^, Dominique Van Praag^139^, Ernest van Veen^64^, Thijs Vande Vyvere^137^, Roel P. J. van Wijk^101^, Alessia Vargiolu^32^, Emmanuel Vega^83^, Kimberley Velt^64^, Jan Verheyden^137^, Paul M. Vespa^140^, Anne Vik^123, 141^, Rimantas Vilcinis^132^, Victor Volovici^67^, Nicole von Steinbüchel^38^, Daphne Voormolen^64^, Petar Vulekovic^46^, Kevin K.W. Wang^142^, Daniel Whitehouse^47^, Eveline Wiegers^64^, Guy Williams^47^, Lindsay Wilson^69^, Stefan Winzeck^47^, Stefan Wolf^143^, Zhihui Yang^113^, Peter Ylén^144^, Alexander Younsi^90^, Frederick A. Zeiler^47,145^, Veronika Zelinkova^20^, Agate Ziverte^60^ , Tommaso Zoerle^27^

^1^ Department of Physiology and Pharmacology, Section of Perioperative Medicine and Intensive Care, Karolinska Institutet, Stockholm, Sweden

^2^ János Szentágothai Research Centre, University of Pécs, Pécs, Hungary

^3^ Division of Clinical Neuroscience, Department of Physical Medicine and Rehabilitation, Oslo University Hospital and University of Oslo, Oslo, Norway

^4^ Department of Neurosurgery, University Hospital Northern Norway, Tromso, Norway

^5^ Department of Physical Medicine and Rehabilitation, University Hospital Northern Norway, Tromso, Norway

^6^ Trauma Surgery, Medical University Vienna, Vienna, Austria

^7^ Department of Anesthesiology & Intensive Care, University Hospital Nancy, Nancy, France

^8^ Raymond Poincare hospital, Assistance Publique – Hopitaux de Paris, Paris, France

^9^ Department of Anesthesiology & Intensive Care, S Raffaele University Hospital, Milan, Italy

^10^ Department of Neurosurgery, Radboud University Medical Center, Nijmegen, The Netherlands

^11^ Department of Neurosurgery, University of Szeged, Szeged, Hungary

^12^ International Projects Management, ARTTIC, Munchen, Germany

^13^ Department of Neurology, Neurological Intensive Care Unit, Medical University of Innsbruck, Innsbruck, Austria

^14^ Department of Neurosurgery & Anesthesia & intensive care medicine, Karolinska University Hospital, Stockholm, Sweden

^15^ NIHR Surgical Reconstruction and Microbiology Research Centre, Birmingham, UK

^16^ Anesthesie-Réanimation, Assistance Publique – Hopitaux de Paris, Paris, France

^17^ Department of Anesthesia & ICU, AOU Città della Salute e della Scienza di Torino - Orthopedic and Trauma Center, Torino, Italy

^18^ Department of Neurology, Odense University Hospital, Odense, Denmark

^19^ BehaviourWorks Australia, Monash Sustainability Institute, Monash University, Victoria, Australia

^20^ Department of Public Health, Faculty of Health Sciences and Social Work, Trnava University, Trnava, Slovakia

^21^ Quesgen Systems Inc., Burlingame, California, USA

^22^ Australian & New Zealand Intensive Care Research Centre, Department of Epidemiology and Preventive Medicine, School of Public Health and Preventive Medicine, Monash University, Melbourne, Australia

^23^ Department of Surgery and Perioperative Science, Umeå University, Umeå, Sweden

^24^ Department of Neurosurgery, Medical School, University of Pécs, Hungary and Neurotrauma Research Group, János Szentágothai Research Centre, University of Pécs, Hungary

^25^ Department of Medical Psychology, Universitätsklinikum Hamburg-Eppendorf, Hamburg, Germany

^26^ Brain Physics Lab, Division of Neurosurgery, Dept of Clinical Neurosciences, University of Cambridge, Addenbrooke’s Hospital, Cambridge, UK

^27^ Neuro ICU, Fondazione IRCCS Cà Granda Ospedale Maggiore Policlinico, Milan, Italy

^28^ ANZIC Research Centre, Monash University, Department of Epidemiology and Preventive Medicine, Melbourne, Victoria, Australia

^29^ Department of Neurosurgery, Hospital of Cruces, Bilbao, Spain

^30^ NeuroIntensive Care, Niguarda Hospital, Milan, Italy

^31^ School of Medicine and Surgery, Università Milano Bicocca, Milano, Italy

^32^ NeuroIntensive Care Unit, Department Neuroscience, IRCCS Fondazione San Gerardo dei Tintori, Monza, Italy

^33^Department of Neurosurgery, Medical Faculty RWTH Aachen University, Aachen, Germany

^34^ Department of Anesthesiology and Intensive Care Medicine, University Hospital Bonn, Bonn, Germany

^35^ Department of Anesthesia & Neurointensive Care, Cambridge University Hospital NHS Foundation Trust, Cambridge, UK

^36^ School of Public Health & PM, Monash University and The Alfred Hospital, Melbourne, Victoria, Australia

^37^ Radiology/MRI department, MRC Cognition and Brain Sciences Unit, Cambridge, UK

^38^ Institute of Medical Psychology and Medical Sociology, Universitätsmedizin Göttingen, Göttingen, Germany

^39^ Oxford University Hospitals NHS Trust, Oxford, UK

^40^ Intensive Care Unit, CHU Poitiers, Potiers, France

^41^ University of Manchester NIHR Biomedical Research Centre, Critical Care Directorate,  Salford Royal Hospital NHS Foundation Trust, Salford, UK

^42^ Movement Science Group, Faculty of Health and Life Sciences, Oxford Brookes University, Oxford, UK

^43^ Department of Neurosurgery, Antwerp University Hospital and University of Antwerp, Edegem, Belgium

^44^ Department of Anesthesia & Intensive Care, Maggiore Della Carità Hospital, Novara, Italy

^45^ Department of Neurosurgery, University Hospitals Leuven, Leuven, Belgium

^46^ Department of Neurosurgery, Clinical centre of Vojvodina, Faculty of Medicine, University of Novi Sad, Novi Sad, Serbia

^47^ Division of Anaesthesia, University of Cambridge, Addenbrooke’s Hospital, Cambridge, UK

^48^ Center for Stroke Research Berlin, Charité – Universitätsmedizin Berlin, corporate member of Freie Universität Berlin, Humboldt-Universität zu Berlin, and Berlin Institute of Health, Berlin, Germany

^49^ Intensive Care Unit, CHR Citadelle, Liège, Belgium

^50^ Department of Anaesthesiology and Intensive Therapy, University of Pécs, Pécs, Hungary

^51^ Departments of Neurology, Clinical Neurophysiology and Neuroanesthesiology, Region Hovedstaden Rigshospitalet, Copenhagen, Denmark

^52^ National Institute for Stroke and Applied Neurosciences, Faculty of Health and Environmental Studies, Auckland University of Technology, Auckland, New Zealand

^53^ Department of Neurology, Erasmus MC, Rotterdam, the Netherlands

^54^ Department of Anesthesiology and Intensive care, University Hospital Northern Norway, Tromso, Norway

^55^ Department of Neurosurgery, Hadassah-hebrew University Medical center, Jerusalem, Israel

^56^ Fundación Instituto Valenciano de Neurorrehabilitación (FIVAN), Valencia, Spain

^57^ Department of Neurosurgery, Shanghai Renji hospital, Shanghai Jiaotong University/school of medicine, Shanghai, China

^58^ Karolinska Institutet, INCF International Neuroinformatics Coordinating Facility, Stockholm, Sweden

^59^ Emergency Department, CHU, Liège, Belgium

^60^ Neurosurgery clinic, Pauls Stradins Clinical University Hospital, Riga, Latvia

^61^ Department of Computing, Imperial College London, London, UK

^62^ Department of Neurosurgery, Hospital Universitario 12 de Octubre, Madrid, Spain

^63^ Department of Anesthesia, Critical Care and Pain Medicine, Medical University of Vienna, Austria

^64^ Department of Public Health, Erasmus Medical Center-University Medical Center, Rotterdam, The Netherlands

^65^ College of Health and Medicine, Australian National University, Canberra, Australia

^66^ Department of Neurosurgery, Neurosciences Centre & JPN Apex trauma centre, All India Institute of Medical Sciences, New Delhi-110029, India

^67^ Department of Neurosurgery, Erasmus MC, Rotterdam, the Netherlands

^68^ Department of Neurosurgery, Oslo University Hospital, Oslo, Norway

^69^ Division of Psychology, University of Stirling, Stirling, UK

^70^ Division of Neurosurgery, Department of Clinical Neurosciences, Addenbrooke’s Hospital & University of Cambridge, Cambridge, UK

^71^ Department of Neurology, University of Groningen, University Medical Center Groningen, Groningen, Netherlands

^72^ Neurointensive Care , Sheffield Teaching Hospitals NHS Foundation Trust, Sheffield, UK

^73^ Salford Royal Hospital NHS Foundation Trust Acute Research Delivery Team, Salford, UK

^74^ Department of Intensive Care and Department of Ethics and Philosophy of Medicine, Erasmus Medical Center, Rotterdam, The Netherlands

^75^ Department of Clinical Neuroscience, Neurosurgery, Umeå University, Umeå, Sweden

^76^ Hungarian Brain Research Program - Grant No. KTIA_13_NAP-A-II/8, University of Pécs, Pécs, Hungary

^77^ Department of Anaesthesiology, University Hospital of Aachen, Aachen, Germany

^78^ Cyclotron Research Center , University of Liège, Liège, Belgium

^79^ Centre for Urgent and Emergency Care Research (CURE), Health Services Research Section, School of Health and Related Research (ScHARR), University of Sheffield, Sheffield, UK

^80^ Emergency Department, Salford Royal Hospital, Salford UK

^81^ Institute of Research in Operative Medicine (IFOM), Witten/Herdecke University, Cologne, Germany

^82^ VP Global Project Management CNS, ICON, Paris, France

^83^ Department of Anesthesiology-Intensive Care, Lille University Hospital, Lille, France

^84^ Department of Neurosurgery, Rambam Medical Center, Haifa, Israel

^85^ Department of Anesthesiology & Intensive Care, University Hospitals Southhampton NHS Trust, Southhampton, UK

^86^ Cologne-Merheim Medical Center (CMMC), Department of Traumatology, Orthopedic Surgery and Sportmedicine, Witten/Herdecke University, Cologne, Germany

^87^ Intensive Care Unit, Southmead Hospital, Bristol, Bristol, UK

^88^ Department of Neurological Surgery, University of California, San Francisco, California, USA

^89^ Department of Anesthesia & Intensive Care,M. Bufalini Hospital, Cesena, Italy

^90^ Department of Neurosurgery, University Hospital Heidelberg, Heidelberg, Germany

^91^ Department of Neurosurgery, The Walton centre NHS Foundation Trust, Liverpool, UK

^92^ Department of Medical Genetics, University of Pécs, Pécs, Hungary

^93^ Department of Neurosurgery, Emergency County Hospital Timisoara , Timisoara, Romania

^94^ School of Medical Sciences, Örebro University, Örebro, Sweden

^95^ Institute for Molecular Medicine Finland, University of Helsinki, Helsinki, Finland

^96^ Analytic and Translational Genetics Unit, Department of Medicine; Psychiatric & Neurodevelopmental Genetics Unit, Department of Psychiatry; Department of Neurology, Massachusetts General Hospital, Boston, MA, USA

^97^ Program in Medical and Population Genetics; The Stanley Center for Psychiatric Research, The Broad Institute of MIT and Harvard, Cambridge, MA, USA

^98^ Department of Radiology, University of Antwerp, Edegem, Belgium

^99^ Department of Anesthesiology & Intensive Care, University Hospital of Grenoble, Grenoble, France

^100^ Department of Anesthesia & Intensive Care, Azienda Ospedaliera Università di Padova, Padova, Italy

^101^ Dept. of Neurosurgery, Leiden University Medical Center, Leiden, The Netherlands and Dept. of Neurosurgery, Medical Center Haaglanden, The Hague, The Netherlands

^102^ Department of Neurosurgery, Helsinki University Central Hospital

^103^ Division of Clinical Neurosciences, Department of Neurosurgery and Turku Brain Injury Centre, Turku University Hospital and University of Turku, Turku, Finland

^104^ Department of Anesthesiology and Critical Care, Pitié -Salpêtrière Teaching Hospital, Assistance Publique, Hôpitaux de Paris and University Pierre et Marie Curie, Paris, France

^105^ Neurotraumatology and Neurosurgery Research Unit (UNINN), Vall d'Hebron Research Institute, Barcelona, Spain

^106^ Department of Neurosurgery, Kaunas University of technology and Vilnius University, Vilnius, Lithuania

^107^ Department of Neurosurgery, Rezekne Hospital, Latvia

^108^ Department of Anaesthesia, Critical Care & Pain Medicine NHS Lothian & University of Edinburg, Edinburgh, UK

^109^ Director, MRC Biostatistics Unit, Cambridge Institute of Public Health, Cambridge, UK

^110^ Department of Physical Medicine and Rehabilitation, Oslo University Hospital/University of Oslo, Oslo, Norway

^111^ Division of Orthopedics, Oslo University Hospital, Oslo, Norway

^112^ Institue of Clinical Medicine, Faculty of Medicine, University of Oslo, Oslo, Norway

^113^ Broad Institute, Cambridge MA Harvard Medical School, Boston MA, Massachusetts General Hospital, Boston MA, USA

^114^ National Trauma Research Institute, The Alfred Hospital, Monash University, Melbourne, Victoria, Australia

^115^ Department of Neurosurgery, Odense University Hospital, Odense, Denmark

^116^ International Neurotrauma Research Organisation, Vienna, Austria

^117^ Klinik für Neurochirurgie, Klinikum Ludwigsburg, Ludwigsburg, Germany

^118^ Division of Biostatistics and Epidemiology, Department of Preventive Medicine, University of Debrecen, Debrecen, Hungary

^119^ Department Health and Prevention, University Greifswald, Greifswald, Germany

^120^ Department of Anaesthesiology and Intensive Care, AUVA Trauma Hospital, Salzburg, Austria

^121^ Department of Neurology, Elisabeth-TweeSteden Ziekenhuis, Tilburg, the Netherlands

^122^ Department of Neuroanesthesia and Neurointensive Care, Odense University Hospital, Odense, Denmark

^123^ Department of Neuromedicine and Movement Science, Norwegian University of Science and Technology, NTNU, Trondheim, Norway

^124^ Department of Physical Medicine and Rehabilitation, St.Olavs Hospital, Trondheim University Hospital, Trondheim, Norway

^125^ Department of Neurosurgery, University of Pécs, Pécs, Hungary

^126^ Division of Neuroscience Critical Care, John Hopkins University School of Medicine, Baltimore, USA

^127^ Department of Neuropathology, Queen Elizabeth University Hospital and University of Glasgow, Glasgow, UK

^128^ Dept. of Department of Biomedical Data Sciences, Leiden University Medical Center, Leiden, The Netherlands

^129^ Department of Pathophysiology and Transplantation, Milan University, and Neuroscience ICU, Fondazione IRCCS Cà Granda Ospedale Maggiore Policlinico, Milano, Italy

^130^ Department of Radiation Sciences, Biomedical Engineering, Umeå University, Umeå, Sweden

^131^ Perioperative Services, Intensive Care Medicine and Pain Management, Turku University Hospital and University of Turku, Turku, Finland

^132^ Department of Neurosurgery, Kaunas University of Health Sciences, Kaunas, Lithuania

^133^ Intensive Care and Department of Pediatric Surgery, Erasmus Medical Center, Sophia Children’s Hospital, Rotterdam, The Netherlands

^134^ Department of Neurosurgery, Kings college London, London, UK

^135^ Neurologie, Neurochirurgie und Psychiatrie, Charité – Universitätsmedizin Berlin, Berlin, Germany

^136^ Department of Intensive Care Adults, Erasmus MC– University Medical Center Rotterdam, Rotterdam, the Netherlands

^137^ icoMetrix NV, Leuven, Belgium

^138^ Movement Science Group, Faculty of Health and Life Sciences, Oxford Brookes University, Oxford, UK

^139^ Psychology Department, Antwerp University Hospital, Edegem, Belgium

^140^ Director of Neurocritical Care, University of California, Los Angeles, USA

^141^ Department of Neurosurgery, St.Olavs Hospital, Trondheim University Hospital, Trondheim, Norway

^142^ Department of Emergency Medicine, University of Florida, Gainesville, Florida, USA

^143^ Department of Neurosurgery, Charité – Universitätsmedizin Berlin, corporate member of Freie Universität Berlin, Humboldt-Universität zu Berlin, and Berlin Institute of Health, Berlin, Germany

^144^ VTT Technical Research Centre, Tampere, Finland

^145^ Section of Neurosurgery, Department of Surgery, Rady Faculty of Health Sciences, University of Manitoba, Winnipeg, MB, Canada

| Åkerlund | Cecilia | cecilia.ai.akerlund@gmail.com |
| --- | --- | --- |
| Amrein | Krisztina | tina.amrein84@gmail.com |
| Andelic | Nada | NADAND@ous-hf.no |
| Andreassen | Lasse | Lasse.Andreassen@unn.no |
| Anke | Audny | [Audny.anke@unn.no](mailto:Audny.anke@unn.no) |
| Antoni | Anna | [anna.antoni@meduniwien.ac.at](mailto:anna.antoni@meduniwien.ac.at) |
| Audibert | Gérard | g.audibert@chu-nancy.fr |
| Azouvi | Philippe | philippe.azouvi@rpc.aphp.fr |
| Azzolini | Maria Luisa | [azzolini.marialuisa@hsr.it](mailto:azzolini.marialuisa@hsr.it) |
| Bartels | Ronald | Ronald.Bartels@radboudumc.nl |
| Barzó | Pál | pbarzo@gmail.com |
| Beauvais | Romuald | beauvais@arttic.eu |
| Beer | Ronny | ronny.beer@i-med.ac.at |
| Bellander | Bo-Michael | bo-michael.bellander@karolinska.se |
| Belli | Antonio | a.belli@bham.ac.uk |
| Benali | Habib | habib.benali@gmail.com |
| Berardino | Maurizio | maurizio_berardino@fastwebnet.it |
| Beretta | Luigi | beretta.luigi@hsr.it |
| Blaabjerg | Morten | [morten.blaabjerg1@rsyd.dk](mailto:morten.blaabjerg1@rsyd.dk) |
| Bragge | Peter | peter.bragge@monash.edu |
| Brazinova | Alexandra | alexandra.brazinova@gmail.com |
| Brinck | Vibeke | vibeke.brinck@quesgen.com |
| Brooker | Joanne | Joanne.Brooker@monash.edu |
| Brorsson | Camilla | [Camilla.Brorsson@umu.se](mailto:Camilla.Brorsson@umu.se) |
| Buki | Andras | 2saturn@gmail.com |
| Bullinger | Monika | bullinger@uke.de |
| Cabeleira | Manuel | mc916@cam.ac.uk |
| Caccioppola | Alessio | alessio.caccioppola@gmail.com |
| Calappi | Emiliana | [calemy02@yahoo.it](mailto:calemy02@yahoo.it) |
| Calvi | Maria Rosa | [calvi.mariarosa@hsr.it](mailto:calvi.mariarosa@hsr.it) |
| Cameron | Peter | [peter.cameron@med.monash.edu.au](mailto:peter.cameron@med.monash.edu.au) |
| Carbayo Lozano | Guillermo | guillermobilbo@gmail.com |
| Carbonara | Marco | marco.carbonara@gmail.com |
| Castaño-León | Ana M. | [ana.maria.castano.leon@gmail.com](mailto:ana.maria.castano.leon@gmail.com) |
| Cavallo | Simona | cavallosimona1@gmail.com |
| Chevallard | Giorgio | [giorgio.chevallard@ospedaleniguarda.it](mailto:giorgio.chevallard@ospedaleniguarda.it) |
| Chieregato | Arturo | [arturo.chieregato@ospedaleniguarda.it](mailto:arturo.chieregato@ospedaleniguarda.it) |
| Citerio | Giuseppe | giuseppe.citerio@unimib.it |
| Clusmann | Hans | hclusmann@ukaachen.de |
| Coburn | Mark Steven | mark.coburn@ukbonn.de |
| Coles | Jonathan | jpc44@wbic.cam.ac.uk |
| Cooper | Jamie D. | jamie.cooper@monash.edu |
| Correia | Marta | Marta.Correia@mrc-cbu.cam.ac.uk |
| Čović | Amra | amra.covic@med.uni-goettingen.de |
| Curry | Nicola | [nicola.curry@ouh.nhs.uk](mailto:nicola.curry@ouh.nhs.uk) |
| Czeiter | Endre | endre.czeiter@gmail.com |
| Czosnyka | Marek | mc141@medschl.cam.ac.uk |
| Dahyot-Fizelier | Claire | c.dahyot-fizelier@chu-poitiers.fr |
| Dark | Paul | paul.m.dark@manchester.ac.uk |
| Dawes | Helen | hdawes@brookes.ac.uk |
| De Keyser | Véronique | [veronique.dekeyser@uza.be](mailto:veronique.dekeyser@uza.be) |
| Degos | Vincent | [vincent.degos@aphp.fr](mailto:vincent.degos@aphp.fr) |
| Della Corte | Francesco | dellacorte.f@gmail.com |
| den Boogert | Hugo | Hugo.denBoogert@radboudumc.nl |
| Depreitere | Bart | bart.depreitere@uzleuven.be |
| Đilvesi | Đula | [djuladjilvesi@gmail.com](mailto:djuladjilvesi@gmail.com) |
| Dixit | Abhishek | ad825@cam.ac.uk |
| Donoghue | Emma | emma.donoghue@monash.edu |
| Dreier | Jens | jens.dreier@charite.de |
| Dulière | Guy-Loup | glduliere@gmail.com |
| Ercole | Ari | ae105@cam.ac.uk |
| Esser | Patrick | pesser@brookes.ac.uk |
| Ezer | Erzsébet | ezererzsebet@yahoo.com |
| Fabricius | Martin | fabricius@dadlnet.dk |
| Feigin | Valery L. | [valery.feigin@aut.ac.nz](mailto:valery.feigin@aut.ac.nz) |
| Foks | Kelly | k.foks@erasmusmc.nl |
| Frisvold | Shirin | Shirin.Kordasti@unn.no |
| Furmanov | Alex | alexpuil@yahoo.com |
| Gagliardo | Pablo | pablog@fivan.org |
| Galanaud | Damien | galanaud@gmail.com |
| Gantner | Dashiell | dashiell.gantner@monash.edu |
| Gao | Guoyi | gao3@sina.com |
| George | Pradeep | george@incf.org |
| Ghuysen | Alexandre | [A.Ghuysen@chu.ulg.ac.be](mailto:A.Ghuysen@chu.ulg.ac.be) |
| Giga | Lelde | lelde.giga@inbox.lv |
| Glocker | Ben | [b.glocker@imperial.ac.uk](mailto:b.glockert@imperial.ac.uk) |
| Golubović | Jagoš | [jagosgolubovic@gmail.com](mailto:jagosgolubovic@gmail.com) |
| Gomez | Pedro A. | [pagolopez@gmail.com](mailto:pagolopez@gmail.com) |
| Gratz | Johannes | johannes.gratz@meduniwien.ac.at |
| Gravesteijn | Benjamin | b.gravesteijn@erasmusmc.nl |
| Grossi | Francesca | francesca.grossi@libero.it |
| Gruen | Russell L. | [russell.gruen@anu.edu.au](mailto:russell.gruen@anu.edu.au) |
| Gupta | Deepak | drdeepakgupta@gmail.com |
| Haagsma | Juanita A. | j.haagsma@erasmusmc.nl |
| Haitsma | Iain | i.haitsma@erasmusmc.nl |
| Helbok | Raimund | Raimund.Helbok@tirol-kliniken.at |
| Helseth | Eirik | EHELSETH@ous-hf.no |
| Horton | Lindsay | [lindsay.horton@stir.ac.uk](mailto:lindsay.horton@stir.ac.uk) |
| Huijben | Jilske | [j.a.huijben@erasmusmc.nl](mailto:j.a.huijben@erasmusmc.nl) |
| Hutchinson | Peter J. | pjah2@cam.ac.uk |
| Jacobs | Bram | [b.jacobs@umcg.nl](mailto:b.jacobs@umcg.nl) |
| Jankowski | Stefan | Stefan.Jankowski@sth.nhs.uk |
| Jarrett | Mike | mike.jarrett@quesgen.com |
| Jiang | Ji-yao | [jiyaojiang@126.com](mailto:jiyaojiang@126.com) |
| Johnson | Faye | faye.johnson@live.co.uk |
| Jones | Kelly | [kejones@aut.ac.nz](mailto:kejones@aut.ac.nz) |
| Karan | Mladen | mladjokaran@gmail.com |
| Kolias | Angelos G. | angeloskolias@gmail.com |
| Kompanje | Erwin | [erwinkompanje@me.com](mailto:erwinkompanje@me.com) |
| Kondziella | Daniel | Daniel.Kondziella@regionh.dk |
| Kornaropoulos | Evgenios | ek481@cam.ac.uk |
| Koskinen | Lars-Owe | [Lars-Owe.Koskinen@umu.se](mailto:Lars-Owe.Koskinen@umu.se) |
| Kovács | Noémi | kovacs.noemi@pte.hu |
| Lagares | Alfonso | algadoc@yahoo.com |
| Lanyon | Linda | lindal@incf.org |
| Laureys | Steven | [steven.laureys@ulg.ac.be](mailto:steven.laureys@ulg.ac.be) |
| Lecky | Fiona | f.e.lecky@sheffield.ac.uk |
| Ledoux | Didier | dledoux@chu.ulg.ac.be |
| Lefering | Rolf | Rolf.Lefering@uni-wh.de |
| Legrand | Valerie | Valerie.Legrand@iconplc.com |
| Lejeune | Aurelie | aurelie.lejeune@chru-lille.fr |
| Levi | Leon | llevi@rambam.health.gov.il |
| Lightfoot | Roger | Roger.Lightfoot@uhs.nhs.uk |
| Lingsma | Hester | h.lingsma@erasmusmc.nl |
| Maas | Andrew I.R. | [andrew.maas@uza.be](mailto:andrew.maas@uza.be) |
| Maegele | Marc | Marc.Maegele@t-online.de |
| Majdan | Marek | [mmajdan@truni.sk](mailto:mmajdan@truni.sk) |
| Manara | Alex | Alex.Manara@nbt.nhs.uk |
| Manley | Geoffrey | ManleyG@ucsf.edu |
| Maréchal | Hugues | Hugues.Marechal@chrcitadelle.be |
| Martino | Costanza | costmartino74@gmail.com |
| Mattern | Julia | Julia.Mattern@med.uni-heidelberg.de |
| McMahon | Catherine | Catherine.McMahon@thewaltoncentre.nhs.uk |
| Melegh | Béla | bela.melegh@aok.pte.hu |
| Menon | David | dkm13@cam.ac.uk |
| Menovsky | Tomas | [tomas.menovsky@uza.be](mailto:tomas.menovsky@uza.be) |
| Mikolic | Ana | a.mikolic@erasmusmc.nl |
| Misset | Benoit | Benoit.Misset@chuliege.be |
| Muraleedharan | Visakh | visakh@incf.org |
| Murray | Lynnette | lynnette.murray@monash.edu |
| Nair | Nandesh | [nandesh.nair@uza.be](mailto:nandesh.nair@uza.be) |
| Negru | Ancuta | [negruancu@gmail.com](mailto:negruancu@gmail.com) |
| Nelson | David | david.nelson@karolinska.se |
| Newcombe | Virginia | vfjn2@cam.ac.uk |
| Nieboer | Daan | [d.nieboer@erasmusmc.nl](mailto:d.nieboer@erasmusmc.nl) |
| Nyirádi | József | nyiradi.jozsef@pte.hu |
| Oresic | Matej | [matej.oresic@oru.se](mailto:matej.oresic@oru.se) |
| Ortolano | Fabrizio | [lupeda@gmail.com](mailto:lupeda@gmail.com) |
| Otesile | Olubukola | o.otesile@sheffield.ac.uk |
| Palotie | Aarno | aarno.palotie@helsinki.fi |
| Parizel | Paul M. | paul.parizel@uantwerpen.be |
| Payen | Jean-François | Jean-Francois.Payen@ujf-grenoble.fr |
| Perera | Natascha | perera@arttic.eu |
| Perlbarg | Vincent | vincent.perlbarg@gmail.com |
| Persona | Paolo | ppersona75@gmail.com |
| Peul | Wilco | W.C.Peul@lumc.nl |
| Piippo-Karjalainen | Anna | anna.piippo@hus.fi |
| Pirinen | Matti | matti.pirinen@helsinki.fi |
| Pisica | Dana | d.pisica@erasmusmc.nl |
| Ples | Horia | horia.ples@neuromed.ro |
| Polinder | Suzanne | s.polinder@erasmusmc.nl |
| Pomposo | Inigo | inigo.pomposo@osakidetza.net |
| Posti | Jussi P. | [jussi.posti@tyks.fi](mailto:jussi.posti@tyks.fi) |
| Puybasset | Louis | louis.puybasset@aphp.fr |
| Rădoi | Andreea | [aradoi@neurotrauma.net](mailto:aradoi@neurotrauma.net) |
| Ragauskas | Arminas | telematics@ktu.lt |
| Raj | Rahul | [rahul.raj@hus.fi](mailto:rahul.raj@hus.fi) |
| Rambadagalla | Malinka | malinka.rambadagalla@gmail.com |
| Rehorčíková | Veronika | rehorcikova@gmail.com |
| Retel Helmrich | Isabel | i.retelhelmrich@erasmusmc.nl |
| Rhodes | Jonathan | jrhodes1@staffmail.ed.ac.uk |
| Richardson | Sylvia | sylvia.richardson@mrc-bsu.cam.ac.uk |
| Richter | Sophie | sr773@cam.ac.uk |
| Ripatti | Samuli | samuli.ripatti@helsinki.fi |
| Rocka | Saulius | saulius.rocka@mf.vu.lt |
| Roe | Cecilie | e.c.t.roe@medisin.uio.no |
| Roise | Olav | olav.roise@medisin.uio.no |
| Rosand | Jonathan | jrosand@partners.org |
| Rosenfeld | Jeffrey | J.Rosenfeld@alfred.org.au |
| Rosenlund | Christina | chrisstenrose@gmail.com |
| Rosenthal | Guy | [rosenthalg@hadassah.org.il](mailto:rosenthalg@hadassah.org.il) |
| Rossaint | Rolf | RRossaint@ukaachen.de |
| Rossi | Sandra | sandrarossi0@gmail.com |
| Rueckert | Daniel | d.rueckert@imperial.ac.uk |
| Rusnák | Martin | mrusnak@igeh.org |
| Sahuquillo | Juan | sahuquillo@neurotrauma.net |
| Sakowitz | Oliver | oliver.sakowitz@gmail.com |
| Sanchez-Porras | Renan | renan_md@hotmail.com |
| Sandor | Janos | sandor.janos@sph.unideb.hu |
| Schäfer | Nadine | Nadine.Schaefer@uni-wh.de |
| Schmidt | Silke | silke.schmidt@uni-greifswald.de |
| Schoechl | Herbert | Herbert.Schoechl@auva.at |
| Schoonman | Guus | g.schoonman@tsz.nl |
| Schou | Rico Frederik | [rico@mymedic.dk](mailto:rico@mymedic.dk) |
| Schwendenwein | Elisabeth | elisabeth.schwendenwein@meduniwien.ac.at |
| Sewalt | Charlie | c.sewalt@erasmusmc.nl |
| Singh | Ranjit | R.D.Singh@lumc.nl |
| Skandsen | Toril | [toril.skandsen@ntnu.no](mailto:toril.skandsen@ntnu.no) |
| Smielewski | Peter | ps10011@cam.ac.uk |
| Sorinola | Abayomi | sorinola_abayomi@hotmail.com |
| Stamatakis | Emmanuel | [eas46@cam.ac.uk](mailto:eas46@cam.ac.uk) |
| Stanworth | Simon | simon.stanworth@nhsbt.nhs.uk |
| Kowark | Ana | akowark@ukaachen.de |
| Stevens | Robert | rstevens@jhmi.edu |
| Stewart | William | [william.stewart@glasgow.ac.uk](mailto:william.stewart@glasgow.ac.uk) |
| Steyerberg | Ewout W. | [e.steyerberg@erasmusmc.nl](mailto:e.steyerberg@erasmusmc.nl) |
| Stocchetti | Nino | stocchet@policlinico.mi.it |
| Sundström | Nina | [Nina.Sundstrom@vll.se](mailto:Nina.Sundstrom@vll.se) |
| Takala | Riikka | [riikka.takala@tyks.fi](mailto:riikka.takala@tyks.fi) |
| Tamás | Viktória | tamas.viktoria@pte.hu |
| Tamosuitis | Tomas | tomas.tamosuitis@kaunoklinikos.lt |
| Taylor | Mark Steven | marktrnava@gmail.com |
| Te Ao | Braden | braden.teao@aut.ac.nz |
| Tenovuo | Olli | olli.tenovuo@tyks.fi |
| Theadom | Alice | alice.theadom@aut.ac.nz |
| Thomas | Matt | Matt.Thomas@nbt.nhs.uk |
| Tibboel | Dick | d.tibboel@erasmusmc.nl |
| Timmers | Marjolijn | mtimmers@hotmail.com |
| Tolias | Christos | christos.tolias@nhs.net |
| Trapani | Tony | tony.trapani@monash.edu |
| Tudora | Cristina Maria | cristina.tudora@neuromed.ro |
| Unterberg | Andreas | Andreas.Unterberg@med.uni-heidelberg.de |
| Vajkoczy | Peter | Peter.Vajkoczy@charite.de |
| Valeinis | Egils | Egils.Valeinis@latnet.lv |
| Vallance | Shirley | S.Vallance@alfred.org.au |
| Vámos | Zoltán | azozoka@gmail.com |
| Van der Jagt | Mathieu | m.vanderjagt@erasmusmc.nl |
| van der Naalt | Joukje | j.van.der.naalt@umcg.nl |
| Van der Steen | Gregory | gregory@webstone.be |
| van Dijck | Jeroen T.J.M. | [j.van.dijck@haaglandenmc.nl](mailto:j.van.dijck@haaglandenmc.nl) |
| van Erp | Inge | i.a.m.van_erp@lumc.nl |
| van Essen | Thomas A. | T.A.van_Essen@lumc.nl |
| Van Hecke | Wim | wim.vanhecke@icometrix.com |
| van Heugten | Caroline | Caroline.vanheugten@maastrichtuniversity.nl |
| Van Praag | Dominique | [dominique.vanpraag@uza.be](mailto:dominique.vanpraag@uza.be) |
| Van Veen | Ernest | e.vanveen.1@erasmusmc.nl |
| van Wijk | Roel | roel-van-wijk@ziggo.nl |
| Vande Vyvere | Thijs | thijs.vandevyvere@icometrix.com |
| Vargiolu | Alessia | neurorianimazione@hsgerardo.org |
| Vega | Emmanuel | emmanuel.vega@chru-lille.fr |
| Velt | Kimberley | [k.velt@erasmusmc.nl](mailto:k.velt@erasmusmc.nl) |
| Verheyden | Jan | jan.verheyden@icometrix.com |
| Vespa | Paul M. | [PVespa@mednet.ucla.edu](mailto:aarno.palotie@helsinki.fi) |
| Vik | Anne | [anne.vik@ntnu.no](mailto:anne.vik@ntnu.no) |
| Vilcinis | Rimantas | rimantas.vilcinis@kaunoklinikos.lt |
| Volovici | Victor | v.volovici@erasmusmc.nl |
| von Steinbüchel | Nicole | nvsteinbuechel@med.uni-goettingen.de |
| Voormolen | Daphne | [d.voormolen@erasmusmc.nl](mailto:d.voormolen@erasmusmc.nl) |
| Vulekovic | Petar | pvulekovic@gmail.com |
| Wang | Kevin K.W. | kawangwang17@gmail.com |
| Whitehouse | Daniel | dw555@cam.ac.uk |
| Wiegers | Eveline | e.wiegers@erasmusmc.nl |
| Williams | Guy | gbw1000@wbic.cam.ac.uk |
| Wilson | Lindsay | l.wilson@stir.ac.uk |
| Winzeck | Stefan | sw742@cam.ac.uk |
| Wolf | Stefan | stefan.wolf@charite.de |
| Yang | Zhihui | [zhihuiyang@ufl.edu](mailto:zhihuiyang@ufl.edu) |
| Ylén | Peter | peter.ylen@vtt.fi |
| Younsi | Alexander | alexander.younsi@med.uni-heidelberg.de |
| Zeiler | Frederick A. | [umzeiler@myumanitoba.ca](mailto:umzeiler@myumanitoba.ca) |
| Ziverte | Agate | agate.ziverte@inbox.lv |
| Zoerle | Tommaso | tommaso.zoerle@policlinico.mi.it |
